# Supplementary material for: Co-evolution of HIV Envelope and Apex-Targeting Neutralizing Antibody Lineage Provides Benchmarks for Vaccine Design
Source: Cell Rep. 2018 Jun 13;23(11):3249–61. doi: 10.1016/j.celrep.2018.05.046 (PMC6019700; doi:10.1016/j.celrep.2018.05.046)
Supplement: Document S1. Supplemental Experimental Procedures, Figures S1–S7, and Tables S1 and S2 [file mmc1.pdf]

Cell Reports, Volume *23*

Supplemental Information

Co-evolution of HIV Envelope and Apex-Targeting Neutralizing Antibody Lineage Provides Benchmarks for Vaccine Design

Kimmo Rantalainen, Zachary T. Berndsen, Sasha Murrell, Liwei Cao, Oluwarotimi Omorodion, Jonathan L. Torres, Mengyu Wu, Jeffrey Umotoy, Jeffrey Copps, Pascal Poignard, Elise Landais, James C. Paulson, Ian A. Wilson, and Andrew B. Ward

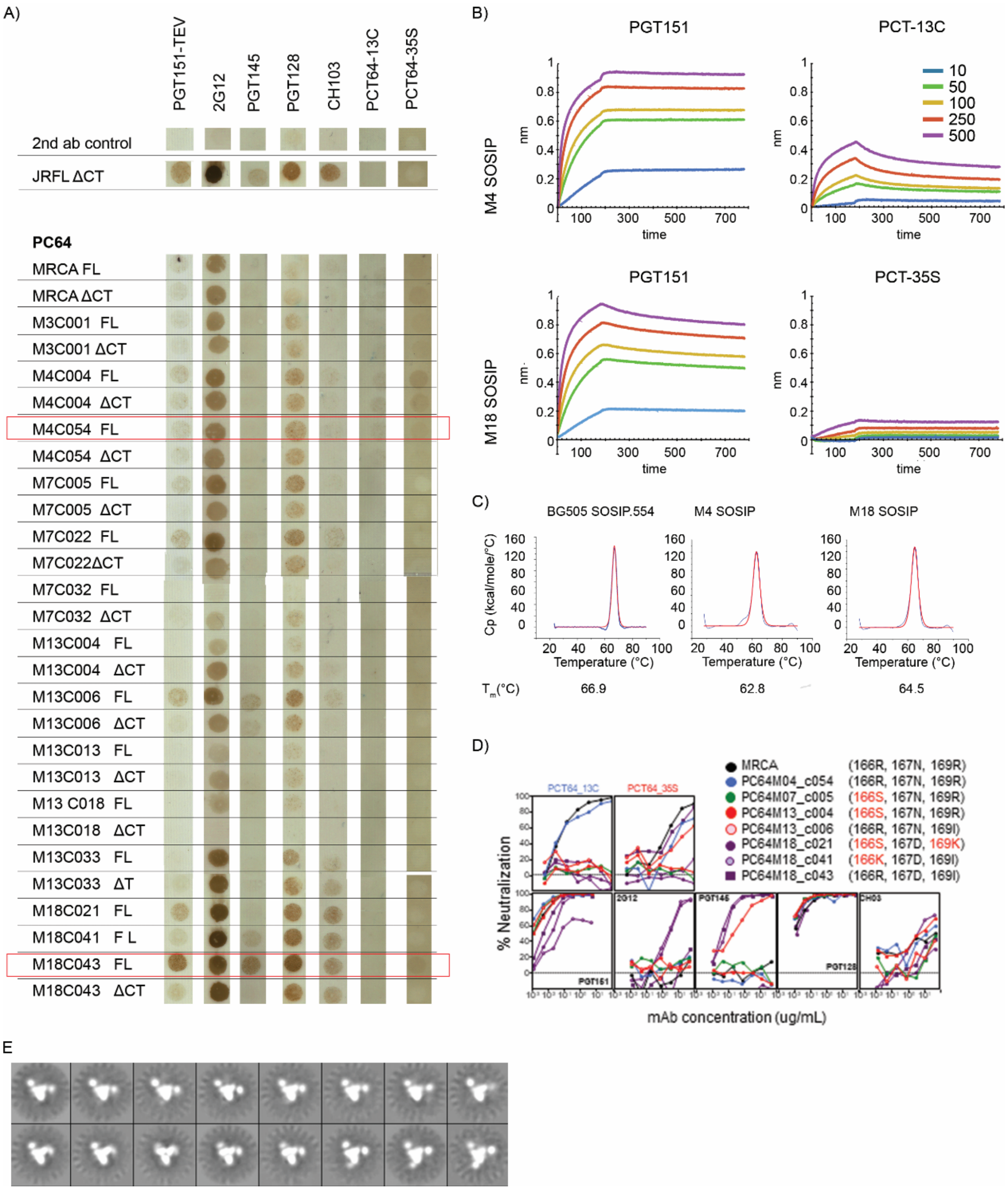

Supplemental Figure 1. **Screening metrics for representative Early and Late timepoint Env and autologous antibodies. Related to Figure 1.** (A) C-terminally truncated (ΔCT) and full-length (FL) Env constructs expression was measured by relative antibody staining in a dotblot assay. Conformationally sensitive control antibodies PGT151 and PGT145 were used to measure expression levels of well-formed trimers. Highest expressing clone PC64M18C043 (Late Env) was selected for high resolution cryo-EM studies and earlier time point clone PC64M4C054 as the representative early Env time point clone. (B) Octet analysis of Early and Late SOSIP binding to PGT151 and autologous Early and Late antibodies. Inset shows the concentration of antibody in nM. (C) Melting point of BG505 SOSIP and PC64 Envs used in this study in a DSC assay. (D) Immunogenic profiling of the selected clones against heterologous antibodies. (E) Negative stain 2D classes of unliganded Late SOSIP.

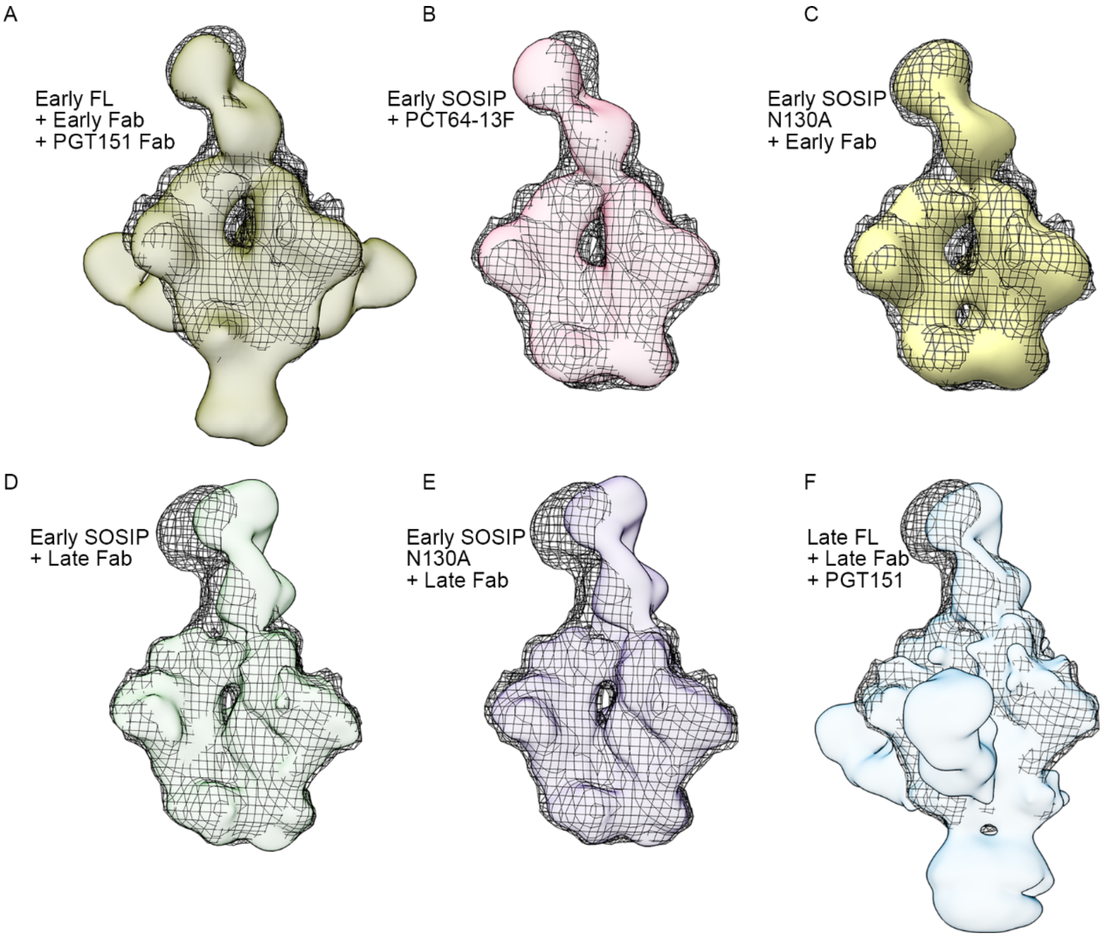

Supplemental Figure 2. Antibody binding angle comparisons. Related to Figures 1 and 2. Autologous complexes are shown in different colors. In each case the Early SOSIP – Early Fab complex (grey mesh) is overlaid as a reference. The binding angle was approx. 20° steeper in all complexes with the Late antibody. A, B and C were reconstructed from negative stain EM data. For comparison, D, E and F were gaussian filtered from higher resolution cryo-EM maps to ~20Å to match the resolution of the negative stain reconstructions.

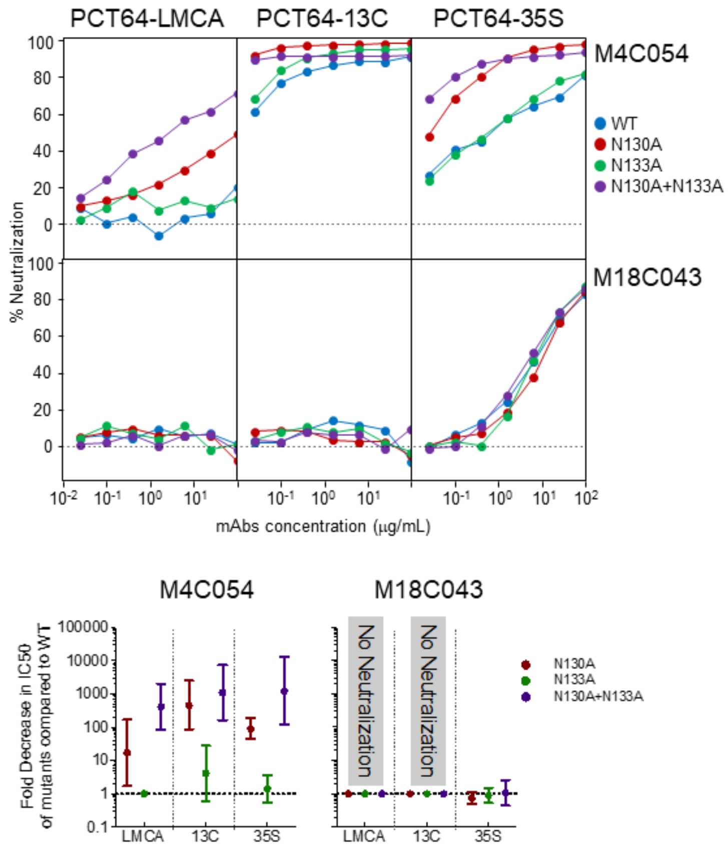

Supplemental Figure 3. Effect of N130- and N133 glycan removal on PC64 early and late autologous neutralization. **Related to Figures 3 and 4.** (A) Autologous neutralization of the WT and mutants Early Env (top) and Late Env (bottom) pseudotyped PC64 viruses by titrated amounts of the indicated PCT64 mAbs, averaged across 8 independent experiments. (B) Geometric mean with geometric SD (N=8) of fold decrease in neutralization IC50 (Ab concentration in μg/mL that represents 50% loss of infectivity) of mutants compared to the corresponding WT virus.

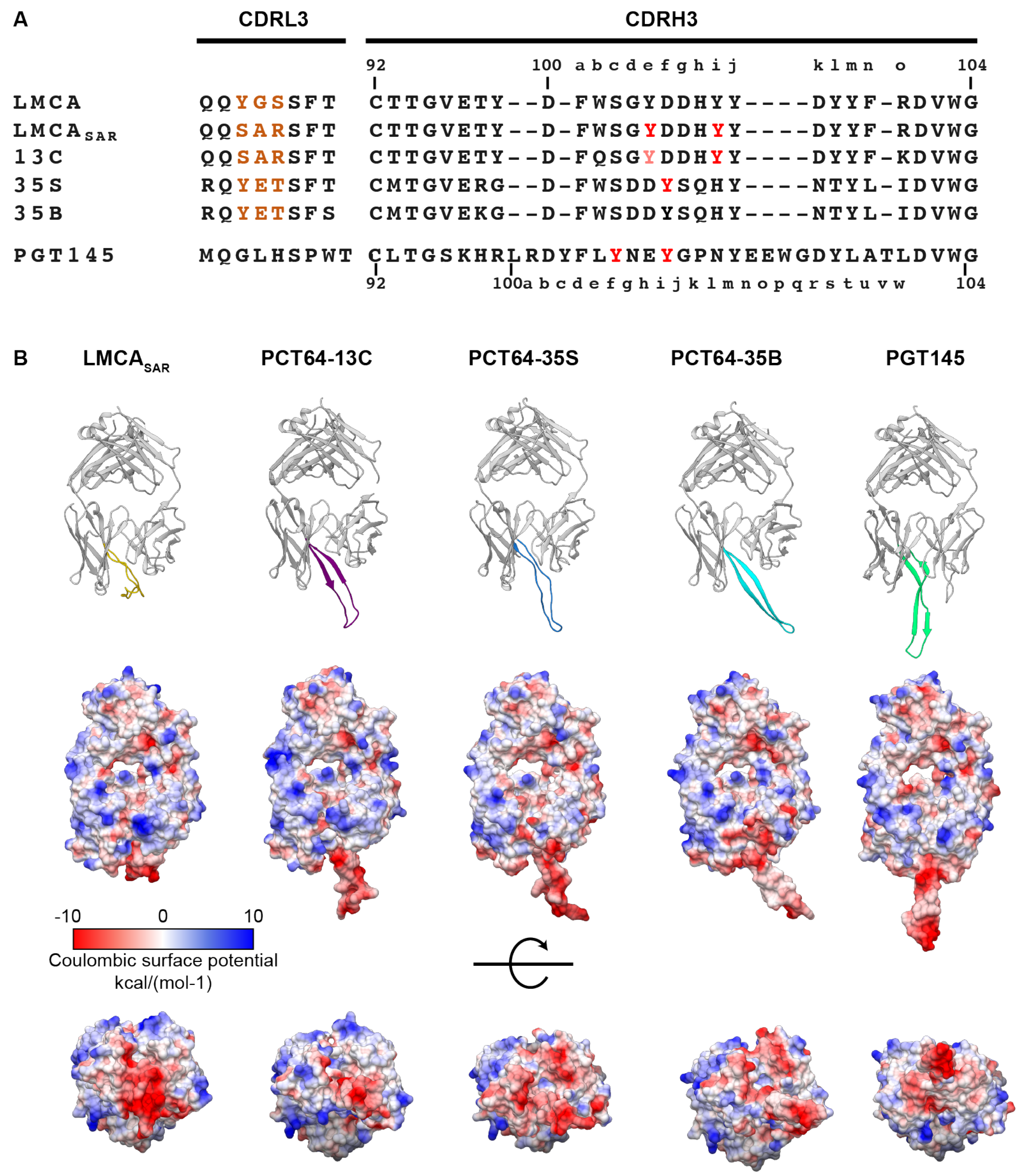

Supplemental Figure 4. Structure and CDR3 sequences of PCT64 and PGT145 antibodies. Related to Figure 1. (A) Alignment of Fab CDRL3 and CDRH3 adapted from T-Coffee (Kabat numbering indicated for CDRH3). Confirmed sulfated tyrosines indicated by red font. (B) Cartoon and Electrostatic surface potential representation of: LMCA$_{SAR}$, PCT64-13C, PCT64-35S, PCT64-35B (5FEH), PGT145 (3U1S).

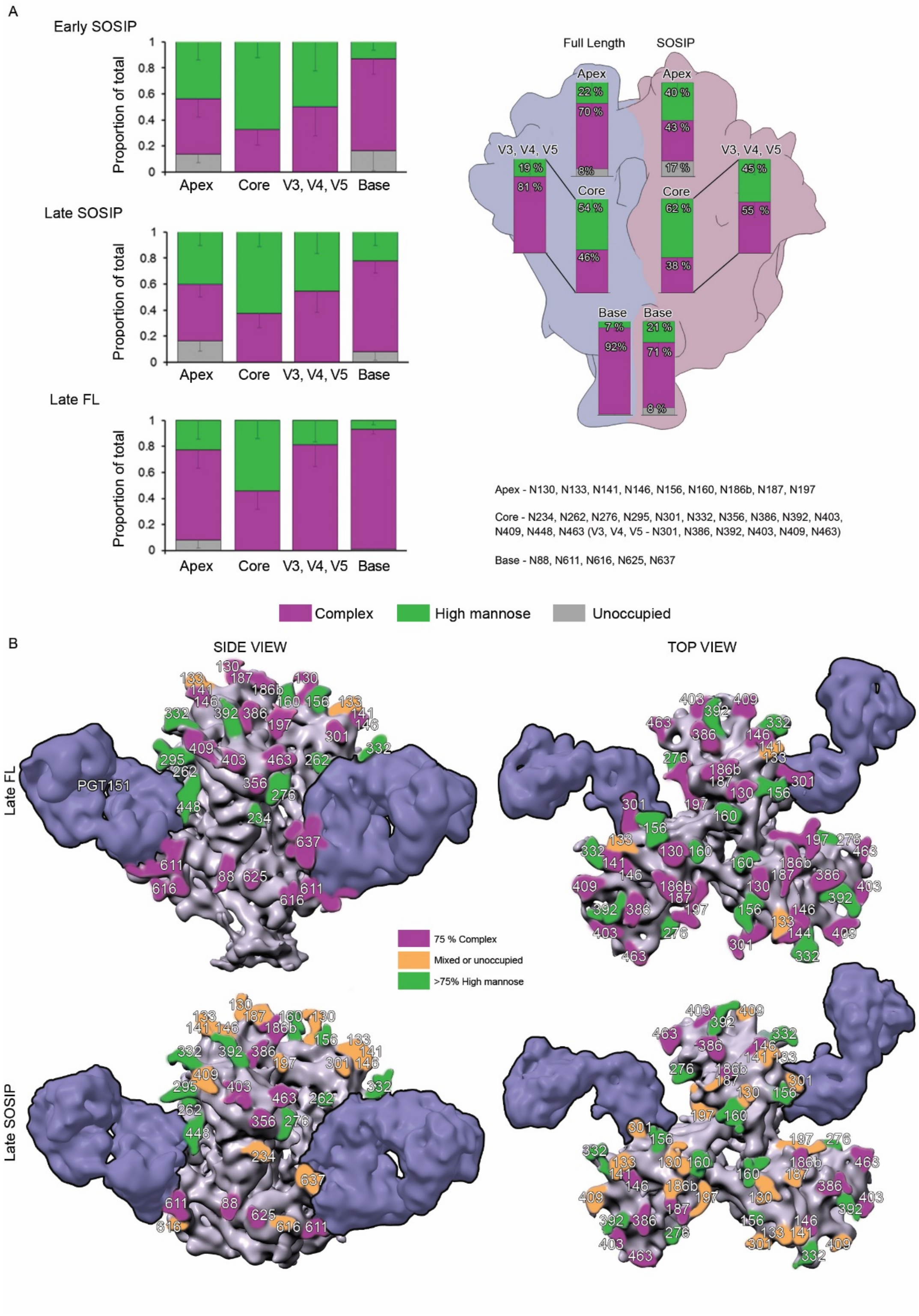

Supplemental Figure 5. Grouping of glycan processing based on their location on Env in FL versus SOSIP. Related to Figure 6. (A) Glycans were grouped into three regions (apex, core, base or V3/V4/V5) based on their location in the trimer as indicated. Proportion of high mannose (green), complex type (purple) or unoccupied (grey) glycosites. (B) Detailed mapping of the glycosylation differences on the surface of corresponding cryo-EM density maps with two PGT151 Fabs bound per trimer.

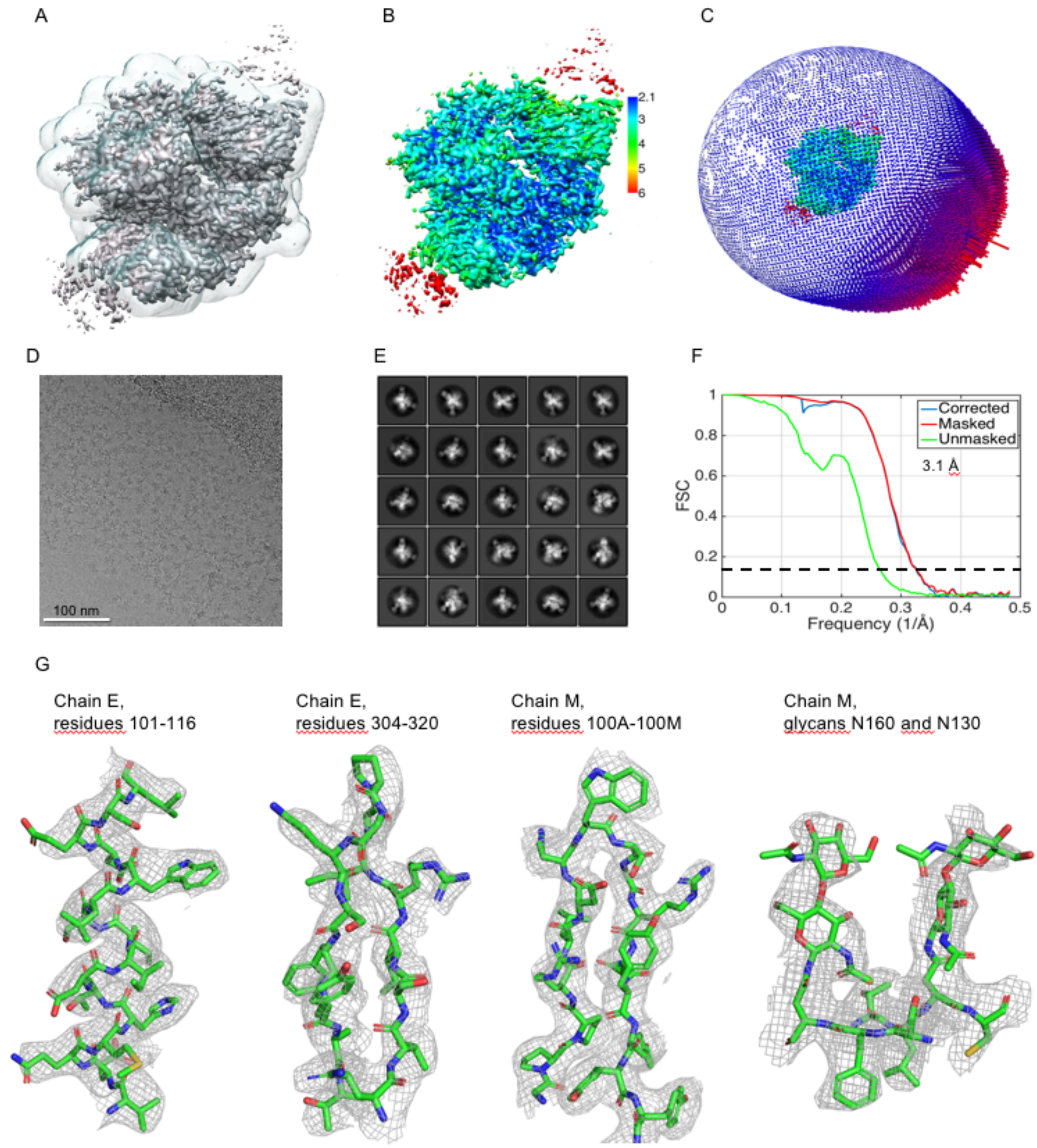

Supplemental Figure 6. Supporting data for Cryo-EM maps. Related to supplemental experimental procedures: EM data processing. (A) Late FL EM density map (grey) with soft binary mask (transparent blue) used during refinement, classification, and sharpening. (B) Local resolution map (ResMap). (C) Anglular distribution 3D histogram. (D) Representative image of aligned raw micrographs. (E) 2D Classes. (F) Fourier-Shell-Correlation (FSC) plot with dashed lined indicating FSC=0.143. (E) Representative density snap shots of the Late FL map.

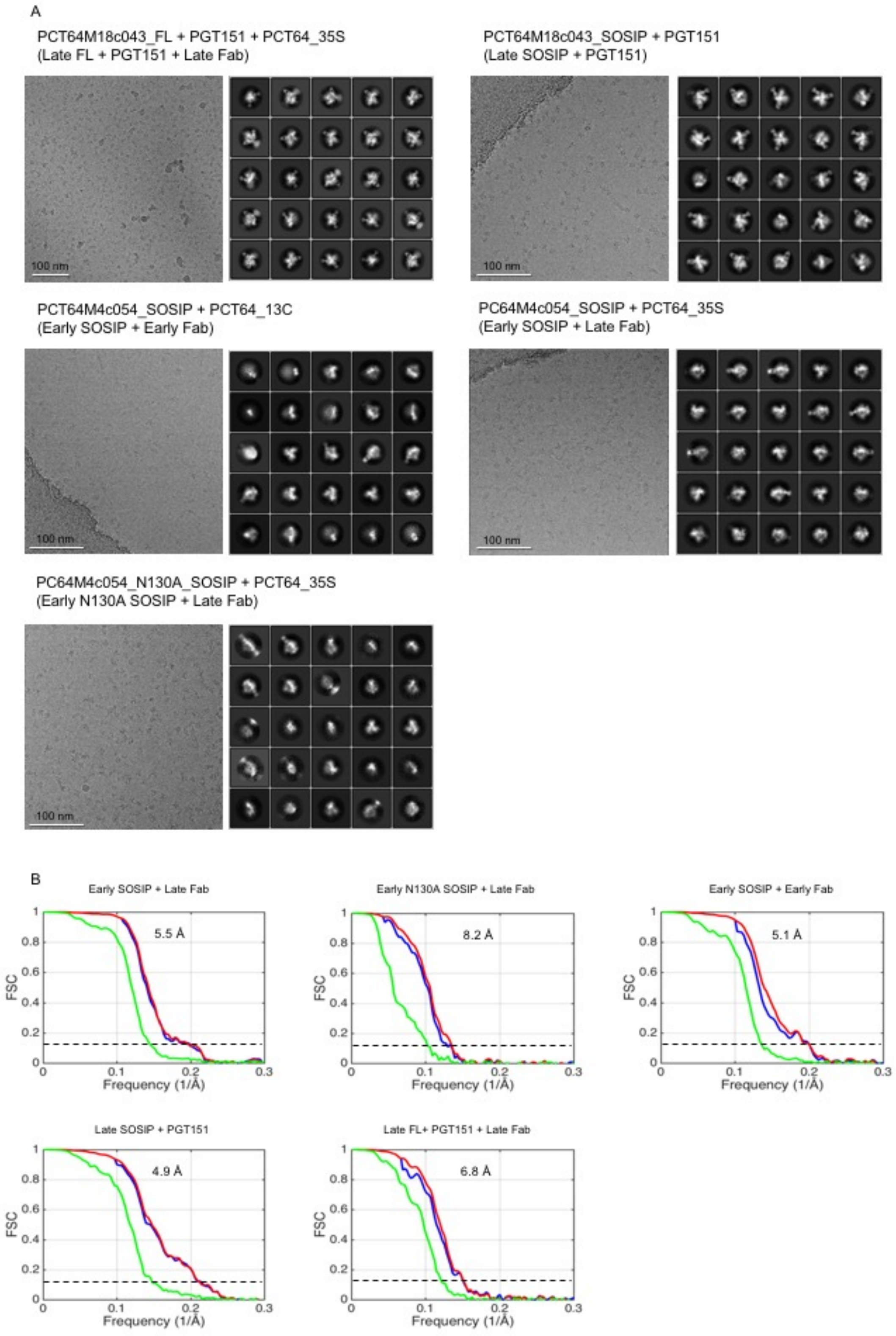

Supplemental Figure 7. Supporting data for Cryo-EM maps. Related to supplemental experimental procedures: EM data processing. (A) Representative images of aligned raw micrographs and 2D classes. (B) FSC plots for 5 cryo-EM maps.

SUPPLEMENTAL FIGURES AND TABLES

Supplemental Table 1. Cryo-EM samples and negative stain, imaging conditions and processing statistics. Related to Figure 1.

**Env**	PC64M18C043 FL (Late FL)	PC64M18C043 FL (Late FL)	PC64M18C043 SOSIP (Late SOSIP)	PC64M4C054 SOSIP (Early SOSIP)	PC64M4C054 SOSIP (Early SOSIP)	PC64M4C054 N130A SOSIP (Early N130A SOSIP)	PC64M4C054 (Early FL)	PC64M4C054 N130A SOSIP (Early N130A SOSIP)	PC64M4C054 (Early SOSIP)
**Ligands**	+ PGT151 Fab	+ PGT151 Fab<br>+ PCT64-35S (Late Fab)	+ PGT151 Fab	+ PCT-13C Fab (Early Fab)	+PCT-35S Fab (Late Fab)	+PCT-35S Fab (Late Fab)	+ PGT151 Fab + PCT13C Fab (Early Fab)	+PCT13C Fab(Early Fab)	+PCT13F Fab
**Sample type**	Cryo-EM	Cryo-EM Cont. Carbon	Cryo-EM	Cryo-EM	Cryo-EM	Cryo-EM	Negative Stain EM	Negative Stain EM	Negative Stain EM
**Data collection**									
Microscope	FEI Titan Krios	FEI Talos Arctica	FEI Titan Krios	FEI Titan Krios	FEI Titan Krios	FEI Titan Krios	FEI Tecnai Spirit	FEI Tecnai Spirit	FEI Tecnai Spirit
Voltage (kV)	300	200	300	300	300	300	120	120	120
Detector	Gatan K2 Summit	Gatan K2 Summit	Gatan K2 Summit	Gatan K2 Summit	Gatan K2 Summit	Gatan K2 Summit	TemCam F416	TemCam F416	TemCam F416
Recording mode	counting	counting	counting	counting	counting	counting	linear	linear	linear
Magnification (incl. post-magnification)	48,534	34,247	48,534	48,534	48,534	48,534	74,146	74,146	74,146
Movie micrograph pixel size (Å)	1.03	1.46	1.03	1.03	1.03	1.03	2.05	2.05	2.05
Dose rate (e^-/[(camera pixel)*s])	10	10	10	10	7.2	8	420	323	323

Number of frames per movie micrograph	40	32	43	43	38	33	NA	NA	NA
Frame exposure time (ms)	200	250	200	200	250	250	250	325	325
Movie micrograph exposure time (s)	8	8	8.6	8.6	9.5	8.25	NA	NA	NA
Total dose ($e^{-}/Å^{2}$)	75.4	37.5	81.1	81.1	64.5	62.2	25	25	25
Defocus range (μm)	-0.5-3.5um	-0.5-4um	-0.2-0.5um	-0.5-3.5um	-0.5-4um	-0.5-4um	-0.85-2.1um	-0.9-2.7um	-1.0-2.7um
**EM data processing**									
Number of movie micrographs	4039	980	1555	2006	2007	867	776	222	370
Number of molecular projection images in map	23,6179	60,093	34,864	29,672	45,661	25,133	2311	2395	8633
Symmetry	C1	C1	C1	C1	C1	C1	C1	C1	C1
Map resolution (FSC 0.143 (CRYO) or 0.5 (NS); Å)	3.1	6.8	4.9	5.1	5.5	8.2	30	30	30
Map sharpening B-factor ($Å^{2}$)	-70	-427	-186	-250	-235	-568	NA	NA	NA
**EMD accession code**	EMD-7858	EMD-7859	EMD-7860	EMD-7863	EMD-7865	EMD-7866	EMD-7861	EMD-7864	EMD-7862

Supplemental Table 2. Data collection parameters and refinement statistics for the Fab crystal structures and for the PC64M18C043 FL cryo-EM structure. Related to Figure 1.

**Name**	PCT64-LMCA ($CDRL3_{SAR}$)	PCT64-13C (Early Fab)	PCT64-35S (Late Fab)	PC64M18C043 FL (Late FL) +PGT151 Fab
**Data Collection**	SSRL 12-2	APS 23-ID-D	APS 23-ID-D	Titan Krios
Wavelength, Å	0.9795	1.0332	1.0332	n/a
Space group	$P2_12_12_1$	$P2_1$	$P2_12_12_1$	n/a
Unit cell a, b, c (Å)	84.20 87.16 142.57	43.09 68.85 78.50	96.78 98.13 100.56	n/a
α, β, γ (°)	90 90 90	90 94.27 90	90 90 90	n/a
Fabs per ASU	2	1	2	n/a
Resolution (Å)*	50.0 – 2.70 (2.75 – 2.70)	50.0 – 1.64 (1.68 -1.64)	50.0 – 2.43 (2.47– 2.43)	3.1
Completeness*	93.4 (68.7)	99.4 (89.3)	99.7 (95.2)	n/a
Redundancy*	5.9 (3.8)	5.9 (3.7)	11.4 (4.7)	n/a
No. total reflections	162,629	326,819	418,936	n/a
No. unique reflections	27,556 (998)	55,491 (2478)	36,695 (1716)	n/a
I/σ*	12.4 (2.3)	19.6 (2.8)	18.3 (12.3)	n/a
R_{sym}*	0.12 (0.47)	0.10 (0.49)	0.15 (0.62)	n/a
R_{pim}*	0.05 (0.25)	0.04 (0.24)	0.04 (0.26)	n/a
$CC_{1/2}$*	0.98 (0.91)	0.98 (0.82)	0.95 (0.75)	n/a
**Refinement statistics**				
Resolution (Å)	43.6 – 2.70	43.0 - 1.64	49.1 - 2.43	3.1
No. reflections total/R_{free}	27,451/1,340	52,835/2634	34,715/1,872	n/a
$R_{cryst}^{‡}$/$R_{free}^{§}$	24.2/26.8	16.9/19.0	20.0/24.5	n/a
RMSD bond length (Å)	0.002	0.006	0.006	0.008
RMSD bond angles (°)	0.49	0.89	0.87	1.282
Protein atoms/solvent atoms	6769/42	3578/515	6777/180	19390/ n/a
Average B-value ($Å^2$) overall/protein/solvent	75/75/51	25/24/33	40/40/36	n/a
Fab 1 B-value ($Å^2$)	62	24	35	n/a
Fab 2 B-value ($Å^2$)	88	n/a	44	n/a
Wilson B-value ($Å^2$)	53	19	35	n/a
Ramachandran Favored%	97.0	98.4	98.1	91.56
Ramachandran Outliers%	0.0	0.0	0.0	0.09
MolProbity score	1.42	0.98	1.13	1.79
ClashScore	4.79	2.12	3.15	5.41
EMringer score	n/a	n/a	n/a	3.43
PDB ID	6CA9	6CA7	6CA6	6DCQ

* Values in parentheses are for highest-resolution shell.

SUPPLEMENTAL VIDEO LEGENDS

Supplemental Video 1. Related to Figure 1. The effect of PGT151 Fab on quaternary structure of Late SOSIP versus Late FL. Both Cryo-EM models were aligned to high resolution reconstruction of the Late ectodomain. At ~6Å resolution a slight change in the orientation of PGT151 Fab was observed.

Supplemental Video 2. Related to Figure 2. Rigidification of CDRH3 and maturation of the antibody approach angle. Rigidification of CDRH3 is shown by morphing the morph between Early SOSIP complexed with either Early or Late Fab. The corresponding crystal structure with the CDRH3 loop fitted in the cryo-EM reconstruction is simultaneously morphed showing the deeper reach of the later antibody. Concomitantly with this, the angle matures to ~20° steeper.

Supplemental Video 3 Related to Figure 3. Apex glycans in Late FL participating in binding of the Late Fab.

Supplemental Experimental Procedures

Expression and folding screening of full length and ΔCT clones

A dotblot assay was developed for screening the expression of full-length and C-terminally truncated clones. A panel of 28 full-length and C- terminal deletion constructs from donor 64 were screened using bnAbs targeting different regions of Env as probes (Figure S1). 750ng of Env DNA and 187.5ng of Furin DNA was mixed with 2.8µg of PEI MAX in 150µl of OPTI-MEM medium in 24 well plates. Plates were sealed with gas-permeable plate seals, vortexed and incubated for 20 min at room temperature prior to addition of 3ml of 293F suspension cells at 1.3 – 1.5 million cells/ml. Cell density at the point of transfection, expression time and incubation conditions were optimized for 24-well plate format and tested for scalability with 25mL and 1L formats. Transfected cells were grown in +37°C, 80% humidity and 8% CO^2 for three days. Plate shaker was set to 500 rpm for 24 well format and to 130 rpm for 25mL and 1L formats. Cell density at the point of harvest (3 days post transfection) was between 4.5 and 6.5 million cells/ml and cell viability >80% in both 24-well, and up-scaled expressions.

Cells were harvested in 24-well plates for 15 min at 1 000 rcf, washed once with 1 ml cold PBS and either resuspended to cold PBS or lysed with TBS containing 0.5% triton X-100 (TBS-T). Suspensions were transferred to 96-well plates to allow application on PVDF membranes with 12-channel pipette. PVDF membranes were pre-wetted with 100% methanol for 20 sec, rinsed briefly with water, and soaked in TBST. Membranes were placed on stack of dry Whatman no. 3 paper followed by a filter paper pre-soaked with TBST to make sure that no air was trapped between the membrane and filter paper. Samples were applied using multichannel pipette with three to five µL of suspension per dot, followed by 20-minute incubation. Membranes were then blocked with PBS containing 5% (w/v) milk powder for 30 minutes in room temperature. Primary antibody was added at 1:1000 – 1:20 000 dilution, depending on the primary antibody, and incubated for 1h in room temperature. Membranes were then washed 3 times 5 minutes with PBS + 0.1% tween-20 (PBST) before the addition of secondary antibody (anti-human IgG, HRP conjugated) and incubated 1h at room temperature, followed by washing 3 times 5 minutes with PBST. Detection was done using colorimetric reaction (CN/DAB Substrate Kit, Thermo Scientific #34000).

Differential scanning calorimetry (DSC)

The thermal stability of the Early and Late SOSIP constructs was measured using a MicroCal VP-capillary DSC calorimeter (Malvern). SOSIP samples were buffer exchanged into phosphate-buffered saline (PBS, pH 7.4) and adjusted to a final concentration of 0.3 mg/mL. Thermal denaturation of each SOSIP sample was probed at a scan rate of 90°C/hr. Normalization and baseline subtraction were performed prior to data analysis using the MicroCal Automatic Analysis program. A final curve was obtained by fitting the data using a non-two-state model.

Biolayer interferometry (BLI)

Biolayer interferometry was used to assess kinetics of Early and Late SOSIP binding to PGT151 antibody and to autologous Early and Late antibodies. Binding measurements were performed using an Octet RED96 instrument (fortéBio, Pall Life Sciences). Assays were conducted at 25°C or 37°C in solid black 96-well plates (Geiger Bio-One) with constant agitation at 1,000 rpm. Antibody (25 µg/mL) in 1X Kinetics Buffer (PBS pH 7.4, 0.01% [w/v] BSA, 0.002% [v/v] Tween 20) was loaded onto anti-human Fc Capture Biosensors (AHC) to threshold binding (approx. 2 nm), followed by a 60 second baseline acquisition in 1X Kinetics Buffer. A concentration gradient of testing antigen (500 nM, 250 nM, 100 nM, 50 nM, 10 nM) was applied for 180 seconds association, followed by measurement of dissociation in 1X Kinetics Buffer for 600 seconds. Octet data were processed using the fortéBio data acquisition program (v.8.1). Experimental data were fitted for PGT151 and Early antibodies using a global fit 1:1 model to determine K_D values. Baseline drift correction was performed by subtraction of the measurement for a sensor loaded with antibody dipped into 1X Kinetics buffer without antigen.

Neutralization assay

Human embryonic kidney (HEK)-derived 293T and HeLa-derived TZM-bl cells were maintained in complete Dulbecco's Modified Eagle Medium containing high-glucose Dulbecco's Modified Eagle Medium (DMEM, Thermo Fisher), 1X Penicillin-Streptomycin (Thermo Fisher) and 10% fetal bovine serum (Gemini Bio Products) at 37°C / 5% CO2. Monoclonal antibody neutralizing activity was assessed using single round of replication in TZM-bl target cells in the absence of DEAE-dextran, as described previously (Landais et al., PLoS Pathog. 2016). Briefly, WT and mutant pseudoviruses were produced by co-transfection of HEK 293-T with an Env-expressing plasmid and an Env-deficient genomic backbone plasmid (pSG3ΔEnv). Env mutagenesis was performed using the Quikchange site-directed mutagenesis kit (Agilent Technologies).

EM data processing

All direct detector movies were aligned and dose-weighted using MotionCor2 (Zheng et al., 2017) (Figure S6, S7). Contrast transfer function (CTF) parameters were estimated from non-dose weighted micrographs using GCTF (Zhang, 2016). Micrographs with estimated resolutions or CTF fits below a certain threshold were removed from the data set. Projection images of Env were selected from each micrograph automatically with either Relion template picking using either templates from previous data sets or a Gaussian blob, or using DoGPicker (Voss et al., 2009). If the micrographs were collected around the edges of carbon holes, EMHP was used to mask out carbon surfaces and filter particle picks (Berndsen et al., 2017). All subsequent downstream single-particle operations were performed with gpu-accelerated Relion/2.0 (Kimanius et al., 2016). As a first step, 4x binned particles were extracted from dose-weighted micrographs and one to three rounds of reference-free 2D classification were performed followed by subset selection and particle re-centering to select for high-resolution classes and remove false positives. These steps

generally remove 10-20% of initial particle picks. Once the data set was deemed sufficiently clean, unbinned particles were extracted and sent through one initial round of unmasked 3D auto-refinement against a reference cryo-EM map of an unliganded HIV-1 Env. Refined Euler angles were used as a starting point for one or more rounds of 3D classification with limited angular sampling. The number of classes in each round of 3D classification varied substantially depending on the specifics of each data set. Homogeneous subsets of 3D classes were selected and particles were re-centered and re-extracted for a final time. At this point, 3D binary masks were generated from heavily lowpass filtered 3D classes and used for any additional rounds of classification and refinement. 3D auto refinement was performed on the homogeneous subsets of particles selected during 3D classification while applying a binary mask. After 3D auto-refinement, each map was sharpened with the mask used in refinement, commonly applying a B-factor generated automatically in Relion. The 3.1 Å Late FL + PGT151 map was sharpened with an ad hoc B-factor of -70 after auto B-factors were determined to be too low by visual inspection. All B-factors used for sharpening are presented in table S1 and all binary masks are deposited to the EMDB, along with unsharpened half maps and final sharped maps. Additional rounds of 3D classification with restricted sampling were performed if the data set was large or heterogeneous, often using high values of the *tau_fudge* (10-20) parameter in Relion, with higher values used for higher resolution data sets. When resolution gains were no longer achieved through classification or classes were deemed to be homogeneous, a final round of 3D auto refinement followed by sharpening was performed. For the 3.1 Å reconstruction of Late FL, an additional step of per-particle CTF estimation was performed with GCTF. CTF parameters were estimated for each particle and used in subsequent rounds of refinement and classification resulting in a 0.3 Å higher resolution reconstruction than was achieved when using CTF parameters estimated from whole micrographs (Figure S6).

Supplemental video editing

Assembly of videos was started by aligning Cryo-EM reconstructions to high resolution reconstruction of Late FL ectodomain using UCSF Chimera. CDRH3 loops of Early and Late Fabs were adjusted according to their corresponding complexed densities using real space refine function in COOT prior to fitting in Cryo-EM maps using Fit In Map function in UCSF Chimera. Morphs between reconstructions in chimera recorded as separate clips in UCSF chimera and assembled in Adobe premiere Pro (Adobe).

Supplemental references

Zheng, S.Q., Palovcak, E., Armache, J.-P., Verba, K.A., Cheng, Y., and Agard, D.A. (2017). MotionCor2: anisotropic correction of beam-induced motion for improved cryo-electron microscopy. Nat. Methods *14*, 331–332.

Zhang, K. (2016). Gctf: Real-time CTF determination and correction. J Struct Biol *193*, 1–12.

Voss, N.R., Yoshioka, C.K., Radermacher, M., Potter, C.S., and Carragher, B. (2009). DoG Picker and TiltPicker: software tools to facilitate particle selection in single particle electron microscopy. J Struct Biol *166*, 205–213.

Berndsen, Z., Bowman, C., Jang, H., and Ward, A.B. (2017). EMHP: An accurate automated hole masking algorithm for single-particle cryo-EM image processing. Bioinformatics.

Kimanius, D., Forsberg, B.O., Scheres, S.H., and Lindahl, E. (2016). Accelerated cryo-EM structure determination with parallelisation using GPUs in RELION-2. Elife *5*, 19.
