# Supplementary material for: Co-evolution of HIV Envelope and Apex-Targeting Neutralizing Antibody Lineage Provides Benchmarks for Vaccine Design
Source: Cell Rep. 2018 Jun 13;23(11):3249–61. doi: 10.1016/j.celrep.2018.05.046 (PMC6019700; doi:10.1016/j.celrep.2018.05.046)
Supplement: Document S2. Article plus Supplemental Information [file mmc5.pdf]

Cell Reports

Article

Co-evolution of HIV Envelope and Apex-Targeting Neutralizing Antibody Lineage Provides Benchmarks for Vaccine Design

Graphical Abstract

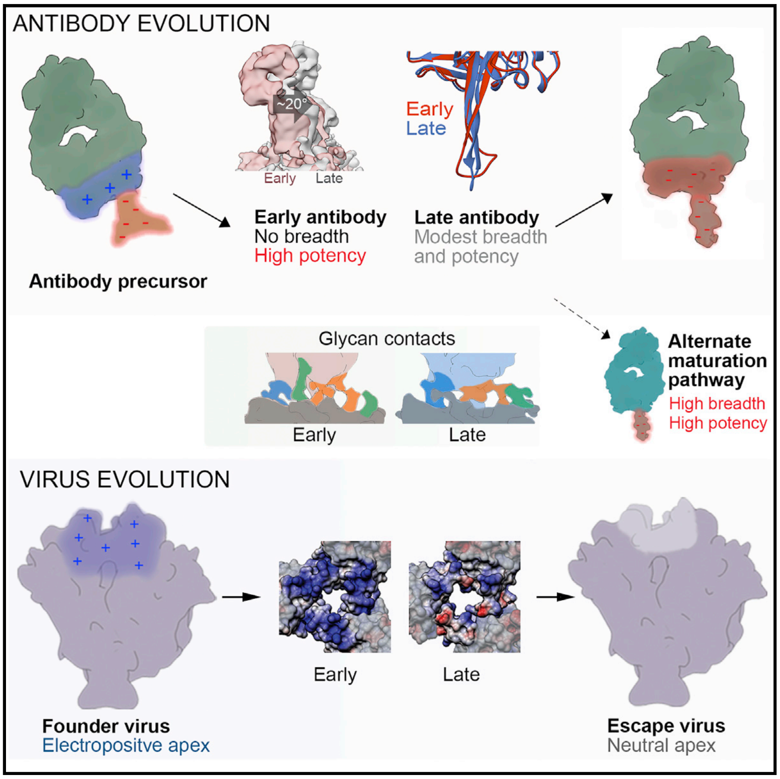

Authors

Kimmo Rantalainen,
Zachary T. Berndsen, Sasha Murrell, ...,
James C. Paulson, Ian A. Wilson,
Andrew B. Ward

Correspondence

andrew@scripps.edu

In Brief

Rantalainen et al. describe the structural co-evolution of HIV envelope glycoprotein and antibody response in a single donor from the Protocol C cohort. The co-evolutionary mechanisms include antibody binding angle maturation, gradual loop rigidification, surface charge modulation, and changes in glycan contacts.

Highlights

- HIV Env-antibody structural co-evolution in PC64 donor involves five key mechanisms
- Antibody binding angle, CDRH3 loop, surface charges, and glycan contacts are affected
- Membrane-bound recombinant Env glycan shield differs from the soluble, SOSIP Env

Data and Software Availability

6CA9
6CA7
6CA6
6DCQ

Rantalainen et al., 2018, Cell Reports *23*, 3249–3261
June 12, 2018 © 2018 The Authors.
https://doi.org/10.1016/j.celrep.2018.05.046

Cell Reports
Article

OPEN ACCESS
CellPress

Co-evolution of HIV Envelope and Apex-Targeting Neutralizing Antibody Lineage Provides Benchmarks for Vaccine Design

Kimmo Rantalainen,[1,4,5] Zachary T. Berndsen,[1,4,5] Sasha Murrell,[1,4] Liwei Cao,[2] Oluwarotimi Omorodion,[1,4] Jonathan L. Torres,[1] Mengyu Wu,[1] Jeffrey Umotoy,[4] Jeffrey Copps,[1,4] Pascal Poignard,[3,4] Elise Landais,[3,4] James C. Paulson,[2] Ian A. Wilson,[1,4] and Andrew B. Ward[1,4,6,*]

[1]Department of Integrative Structural and Computational Biology, The Scripps Research Institute, La Jolla, CA 92037, USA
[2]Department of Molecular Medicine, The Scripps Research Institute, La Jolla, CA 92037, USA
[3]Department of Immunology and Microbial Science, The Scripps Research Institute, La Jolla, CA 92037, USA
[4]IAVI Neutralizing Antibody Center and Collaboration of AIDS Vaccine Discovery, The Scripps Research Institute, La Jolla, CA 92037, USA
[5]These authors contributed equally
[6]Lead Contact
*Correspondence: andrew@scripps.edu
https://doi.org/10.1016/j.celrep.2018.05.046

SUMMARY

Broadly neutralizing antibodies (bnAbs) targeting the HIV envelope glycoprotein (Env) typically take years to develop. Longitudinal analyses of both neutralizing antibody lineages and viruses at serial time points during infection provide a basis for understanding the co-evolutionary contest between HIV and the humoral immune system. Here, we describe the structural characterization of an apex-targeting antibody lineage and autologous clade A viral Env from a donor in the Protocol C cohort. Comparison of Ab-Env complexes at early and late time points reveals that, within the antibody lineage, the CDRH3 loop rigidifies, the bnAb angle of approach steepens, and surface charges are mutated to accommodate glycan changes. Additionally, we observed differences in site-specific glycosylation between soluble and full-length Env constructs, which may be important for tuning optimal immunogenicity in soluble Env trimers. These studies therefore provide important guideposts for design of immunogens that prime and mature nAb responses to the Env V2-apex.

INTRODUCTION

The development of an effective HIV vaccine has presented an enormous challenge over the past 30 years, largely due to the immune evasion mechanisms of the envelope glycoprotein (Env), the sole target for antibody-mediated neutralization. Several regions of Env have now been characterized as epitopes for broadly neutralizing antibodies (bnAbs), including the V2 apex, CD4 receptor binding site (CD4bs), N332 glycan supersite, gp120/gp41 interface, and the membrane proximal region (MPER) (Burton and Hangartner, 2016). Each epitope is protected from the immune system to varying degrees by evasion mechanisms, including rapid evolution of variable loop sequences and length, glycan shielding, and steric barriers (Burton and Hangartner, 2016; Johnson and Desrosiers, 2002). Recent efforts in HIV vaccine development have employed soluble, ectodomain trimers as immunogens in hopes of eliciting cross-clade bnAb responses. This approach has been successful in eliciting autologous neutralizing antibody responses in rabbits, guinea pigs, and non-human primates (Cheng et al., 2015; Dubrovskaya et al., 2017; Feng et al., 2016; Klasse et al., 2016; McCoy et al., 2016; Pauthner et al., 2017; Sanders et al., 2015). Alternative approaches, including epitope focusing and germline targeting, aim to enrich for particular and desirable B cell precursor pools that have been previously identified as producing bnAbs in infected humans. Such approaches are designed to guide the immune system toward breadth via rational boosting strategies (Briney et al., 2016; Escolano et al., 2016; Jardine et al., 2013, 2016; Steichen et al., 2016).

Understanding the co-evolution of the virus and immune response during human infection can provide valuable clues about how the immune system overcomes immune evasion barriers. These insights, in turn, can help guide immunogen design aimed at priming the immune system to preferentially elicit advantageous antibodies via a vaccine. Only a handful of such co-evolution studies have been completed to date, each offering valuable insights into evolution of naturally occurring bnAbs (reviewed in Stamatatos et al., 2017). These analyses thereby define the critical features for acquisition of neutralization breadth and can be used to design sequential immunization regimens that may recapitulate the infection model. We previously characterized the V2-apex PCT64 bnAb lineage from an elite neutralizer in the International AIDS Vaccine Initiative (IAVI) Protocol C (PC) cohort (Landais et al., 2016, 2017). Acquisition of heterologous neutralization breadth in the PCT64 lineage coincided with concomitant changes in complementarity-determining region H2 (CDRH2) and CDRH3 and adaptation to viral escape mutations at V2 loop residues 166 and 169, as previously observed for the CAP256-VRC26 V2 apex bnAb lineage (Doria-Rose et al., 2015). The relatively early emergence of the PCT64

This is an open access article under the CC BY license (http://creativecommons.org/licenses/by/4.0/).

lineage (~7 months post-infection), with low levels of somatic hypermutation (10%–12%), a PGT145-like extended CDRH3 loop conformation of moderate length (25 amino acids [aas]), and the absence of detectable autoreactivity, makes this lineage an attractive target for epitope-targeted immunogen design (Landais et al., 2017).

Structural characterization of Env-antibody complexes has provided some mechanistic details of co-evolution (Bonsignori et al., 2016; Garces et al., 2014) but has so far been limited to heterologous trimeric complexes or autologous monomeric gp120 subunits. In this study, we combined negative stain and cryoelectron microscopy (cryo-EM) single-particle approaches with X-ray crystallographic studies of early and late autologous Env-antibody complexes to decipher molecular details of the co-evolution of HIV Env and the immune system response over the course of several years of chronic infection. In addition to structural studies, mass spectrometric analysis of N-linked glycosylation was performed for membrane-bound and corresponding soluble SOSIP trimers, with notable differences observed in biosynthetic processing of N-linked glycans. Our data show that PCT64 antibodies target Env in a similar way to one of the most broad and potent bnAbs to date, PGT145/PGDM1400 (Lee et al., 2017; Sok et al., 2014; Walker et al., 2011, 2009) but do so using a shorter CDRH3. The pathway of PCT64, however, evolves to a less broad and potent response than PGT145/PGDM1400. The structures of key time points in the PCT64 lineage can now be used as guides to promote responses that are more akin to the superior PGT145/PGM1400 phenotype.

RESULTS

Characterization of Multiple Autologous Complexes of Env and Antibody from a Single Donor

Longitudinal full-length clade A Env clones (N = 98) covering 10 time points across 46 months post-infection were recently described (Landais et al., 2017). In pursuit of a structural understanding of the co-evolution of virus and immune response within a single patient, we screened expression of 28 Env constructs spanning from early to late time points (Supplemental Experimental Procedures; Figure S1A). From our initial screening, a full-length Env clone no. 43 (PC64M18C043) that was isolated from the 18 months post-infection sample (from here on referred to as late Env) showed the highest expression levels in HEK293F cells, as well as a native antigenic profile (Figure S1). This Env clone was subsequently scaled up and purified from the cell surface via PGT151 affinity capture (Blattner et al., 2014; Lee et al., 2016). Notably, PGT151 completely and potently neutralized late Env pseudovirus at 50% inhibitory concentration (IC_{50}) of 0.0013 μg/mL and did not exhibit any neutralization plateau (Figure S1D), suggesting that our approach extracted all functional, native Env from the membrane. From this construct, we obtained a particularly stable complex that resulted in a 3.1 Å resolution cryo-EM reconstruction of full-length late Env bound to PGT151 that enabled us to build and refine an atomic model (Figures 1A and 1B; Table S1). Our late full-length (FL) structure was remarkably similar to the clade B JR-FLΔCT-PGT151 structure (Cα RMSD = 1.58 Å; PDB ID: 5FUU) and to soluble SOSIP structures from other subtypes. Like JR-FLΔCT-PGT151, only the ectodomain, excluding the membrane proximal external region (MPER), was well resolved (Figure 1A). Thus, the presence of the cytoplasmic C-terminal tail (CT) does not appear to influence the conformational stability of the MPER and transmembrane domain (TM), at least not in the detergent micelle milieu. Similar to previous structural studies of Env, the majority of the unliganded glycans could only be resolved between one to three sugar moieties, indicative of a high degree of conformational flexibility in the glycan shield. The same Env clone was solved as a SOSIP.664 trimer (late SOSIP) in complex with PGT151 to 4.9 Å resolution for direct comparison with the FL structure. Similar to FL Env, PGT151 bound to SOSIP trimers with a stoichiometry of two per trimer and induced a subtle conformational change that resulted in opening of one of the protomer interfaces. The only observed difference between the FL and SOSIP complexes was a subtle difference in PGT151 orientation (Video S1).

Guided by neutralization data of autologous antibodies (Landais et al., 2017), we selected a late time point PCT64 Ab to make an autologous complex matching our late Env with a late antibody. This antibody was isolated from the month 35 sample, PCT64-35S (late Fab), and neutralized late M18 virus at an IC_{50} of 9 μg/mL. Despite the relatively poor neutralization, we obtained a stable complex that resulted in a cryo-EM reconstruction of a late FL-late Fab complex bound to PGT151 at 6.8 Å resolution (Figure 1C; Table S1). Notably, the presence of PGT151 did not impact binding of the autologous antibody, confirming that PGT151 does not alter the structure of the apex. The high-resolution structure of the late FL-PGT151 Fab complex described above could be readily docked into the late Fab bound complex, as could a 2.4-Å-resolution crystal structure of late Fab (Figures 1F, 1I, and S4; Table S2), enabling a pseudo-atomic interpretation of the epitope-paratope interactions discussed below. Despite several efforts, we did not observe binding (Figure S1B), nor could we form a complex of the late SOSIP and late Fab, which is somewhat paradoxical as the late FL clone bound the Fab and the associated virus was neutralized by the antibody (Landais et al., 2017). We note that apex-targeting bnAbs required a well-formed trimer to bind, and the instability of the late SOSIP observed in our negative stain electron microscopy (NS-EM) analysis could therefore explain the poor binding properties (Figure S1E). This instability manifested as mainly open trimers dissociating into monomers and dimers during our attempts to prepare late SOSIP complexes. Additionally, as discussed below, differences in the site-specific glycosylation between the two constructs could also influence binding.

To complement these late Env structures and achieve a longitudinal comparison, we also obtained cryo-EM reconstructions of an early Env, PC64M4C054, expressed as a SOSIP.664 trimer (early SOSIP) in complex with both an antibody isolated from month 13, PCT64-13C (early Fab), and the late Fab at 5.1 Å and 5.5 Å resolution, respectively (Figures 1D, 1E, and S2; Table S1). The early Fab was the closest relative to the least mutated common ancestor (LMCA) among the PCT64 monoclonal antibodies and neutralized early M4 virus at an IC_{50} of 0.0229 μg/mL (Figure S1D). We solved crystal structures of both the early and late Fabs at 1.6 Å and 2.4 Å, respectively

OPEN
ACCESS
CellPress

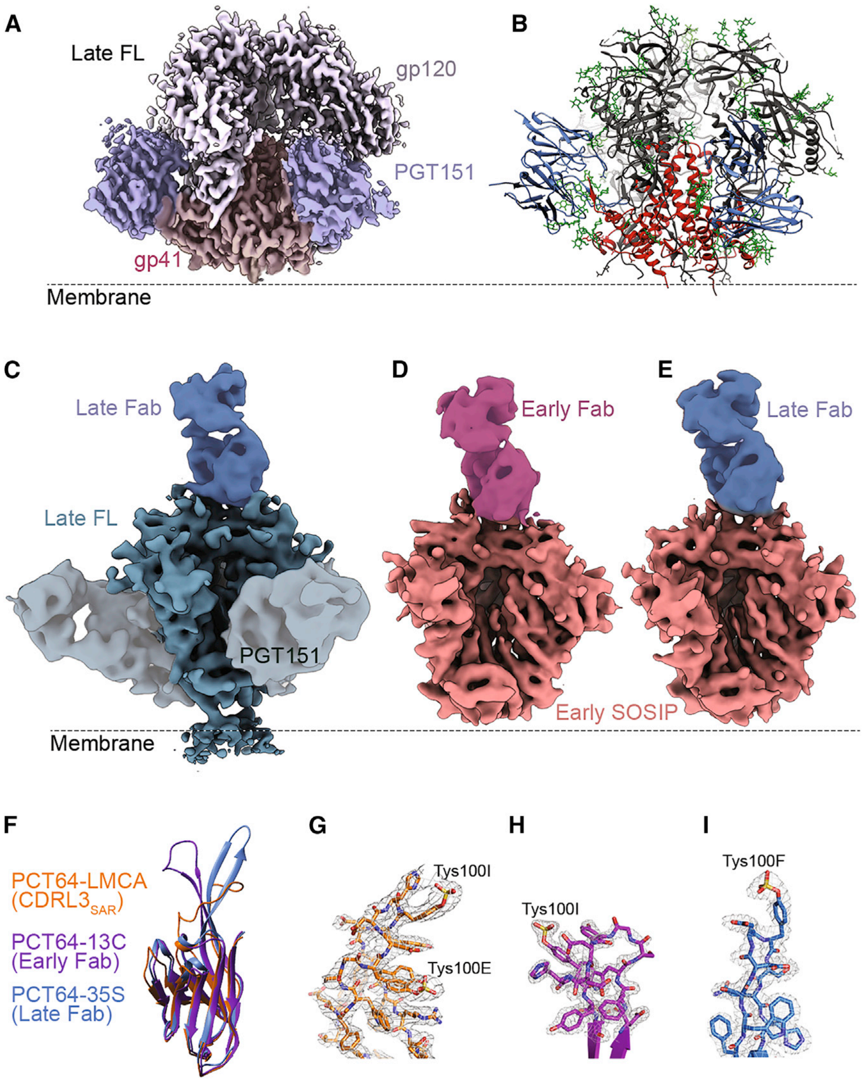

Figure 1. Cryo-EM Structures of PC64 Env and Autologous Antibody Complexes and X-Ray Structures of PCT64 Fabs

(A) Cryo-EM map of the late FL ectodomain with PGT151 bound at ~3.1 Å resolution.

(B) Corresponding atomic structure (gp41 red, gp120 dark gray, PGT151 Fab blue, and glycans depicted as green sticks).

(C) 6.8 Å resolution cryo-EM reconstruction of autologous complex of late FL with late Fab and PGT151.

(D) 5.1 Å resolution cryo-EM reconstruction of autologous complex of early SOSIP with early Fab.

(E) 5.5 Å resolution cryo-EM reconstruction of an autologous complex of early SOSIP with late Fab.

(F) Heavy chain structures of LMCA ($CDRL3_{SAR}$) Fab (orange), early Fab (purple), and late Fab (blue).

(G–I) Close up of the apical residues of the CDRH3 loop for (G) LMCA ($CDRL3_{SAR}$), (H) early, and (I) late Fabs (experimental 2Fo-Fc electron density map shown as a gray mesh [σ = 1.0]).

(Figures 1F, 1H, 1I, and S4; Table S2). Additionally, we solved the 2.7 Å resolution structure of a variant of the LMCA, LMCA ($CDRL3_{SAR}$), where CDRL3 is mutated from the wild-type ^{91}YGS^{93} to ^{91}SAR^{93} (Figures 1F, 1G, and S4; Table S2). These mutations were earlier shown to be critical for acquisition of autologous neutralization by the 13C antibody (Landais et al., 2017). Finally, we obtained a negative stain reconstruction of an early FL-early Fab complex; however, low expression levels of the early FL precluded high-resolution cryo-EM studies (Figures S1A and S2A). Our 3.1 Å resolution model and Fab structures could be docked in the cryo-EM complexes, allowing pseudo-atomic interpretations of the epitope-paratope interactions.

Rigidification of CDRH3 during Co-evolution

The crystal structures of the early and late Fabs both contain a CDRH3 loop that adopts an extended β-hairpin conformation with similar conservation of negatively charged residues, which corresponds with the previously solved X-ray structure of PCT64-35B, also isolated from month 35 (Landais et al., 2017; PDB 5FEH; Figures 1 and S4). The PCT64-35B crystal structure did not contain electron density for a sulfated tyrosine in CDRH3; however, mass spectrometry data suggested the presence of a sulfated residue (Landais et al., 2017). Here, a sulfotyrosine was observed at position 100F of the late Fab CDRH3 (Figure 1I). Whereas it is difficult to directly compare the Fab structure with that of PGT145 due to the difference in the CDRH3 angle, alignment of the CDRH3 hairpin alone demonstrates that the sulfated tyrosine at 100F in the late Fab overlaps with the sulfate at Tyr100I of the PGT145 Fab (McLellan et al., 2011; Figure S4A). Consistent with computational predictions that sulfotyrosines are common in earlier antibodies from this lineage, we observe a sulfated tyrosine at position 100I of the early Fab CDRH3 (Figure 1H; Landais et al., 2017). Due to the disordered residues at the tip of the CDRH3 of the early Fab, we were unable to confirm whether any of the other tyrosines in the region are sulfated. Electrospray ionization (ESI)-mass spectrometry (MS) analysis, however, indicates a heavy chain molecular weight ~160 Da above the predicted mass, suggesting the presence of an additional sulfate in the Fab.

The overall structure of the late Fab was similar to the early Fab, apart from CDRH3, which at the early time point adopts a β-hairpin at the base but is "splayed" at the tip, resulting in a very different paratope (Figures 1F and 1I). In cryo-EM maps of both the early SOSIP and late FL in complex with the late Fab, CDRH3 was sufficiently resolved to allow manual docking of the loop (Figure 2; Video S2). Whereas crystal packing interactions may influence the CDRH3 conformation (MacLeod et al., 2016), we concluded that the splayed

OPEN ACCESS
CellPress

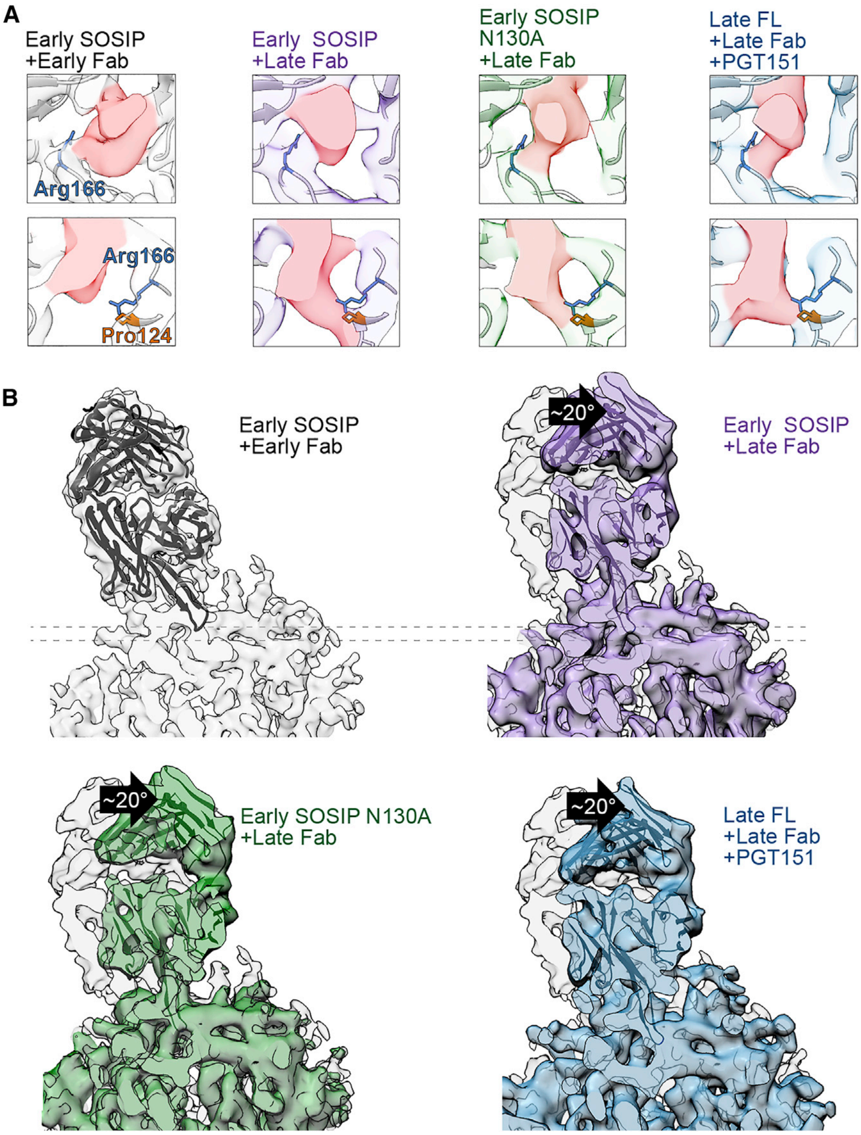

Figure 2. Env Contacts and Binding Angles of Early and Late Antibodies

(A) Cross-sections (dotted lines in B) of trimer three-fold axis. Fab densities are highlighted in red. Early and late Fabs have a conserved contact with Arg166 in protomer 2 (upper row). Late Fab contacts Arg166 in protomer 3 and Pro124 in protomer 2 of Env at ~5 Å deeper into the trimer apex (lower row).

(B) Early SOSIP with early Fab (gray), superimposed with late Fab bound to early SOSIP (purple), to early SOSIP N130A (green), and to late FL and PGT151 (blue) showing an ~20° shift in angle of approach. Fab crystal structures are docked into cryo-EM density maps (ribbon).

conformation of the PCT64-13C was the likely conformation of the unliganded antibody based on two observations: the early Fab contains a Gly100D "hinge" that could allow for splaying, and this conformation is observed in the cryo-EM complex of early SOSIP-early Fab (Figure 1). Gly100D is mutated to aspartic acid by the next time point of sampling (month 18) and is, therefore, most likely one of the contributing mutations toward deeper penetration of the CDRH3 tip into the trimer apex.

The progressive rigidification of the CDRH3 is further illustrated by the crystal structure of the LMCA-CDRL3$_{SAR}$. Here, the entire CDRH3 lacks secondary structure, indicating an even more plastic structure than the early Fab (Figures 1G and S4). Because of the steric barrier comprised of apex glycans, it is unlikely that the LMCA binds with CDRH3 in the collapsed conformation that is observed in the crystal structure. Rather, it is likely that there is induced fit where CDRH3 adopts a more extended conformation upon binding that enables the acidic residues of CDRH3 to interact with the positively charged apex.

Both early and late Fabs make contacts with Arg166 in V2 of gp120 in the cryo-EM maps, but all complexes with the late Fab have an additional contacting density further down the three-fold axis, emanating from the tip of CDRH3 toward the highly conserved Pro124 and Arg166 of the third protomer (Figure 2A; Video S2). This contact is suggestive of an increasing dependency on Arg166 engagement in later time points and in agreement with earlier observations of the critical importance of mutations at this position for viral escape (Landais et al., 2017). Exact residues of the CDRH3 contacting gp120 could not be definitively determined because CDRH3 loops of early and late Fab crystal structures required manual fitting into moderate-resolution cryo-EM maps, also indicating that the CDRH3 beta-hairpin may undergo a subtle induced fit upon binding to Env.

Antibody Maturation Is Accompanied by Steepening of the Angle of Approach

In all our reconstructions of the early Env-early Fab complex, the Fab angle of approach differs from complexes with the late Fab (Figures 2B and S2; Video S2). The early Env-early Fab complex was previously characterized by low-resolution cryo-EM and negative stain analysis (Landais et al., 2017). Here, our improved cryo-EM reconstruction of the early SOSIP-early Fab and a direct comparison to early SOSIP-late Fab confirmed the ~20° steeper binding angle of the late antibody. Strong density emanating from the bound late Fab CDRH3 tip (Figure 2A) differs from BG505 SOSIP bound to PGT145, where Tyr100I is observed in the middle of the three-fold axis (Lee et al., 2017). No effect on the relative protomer positions was observed

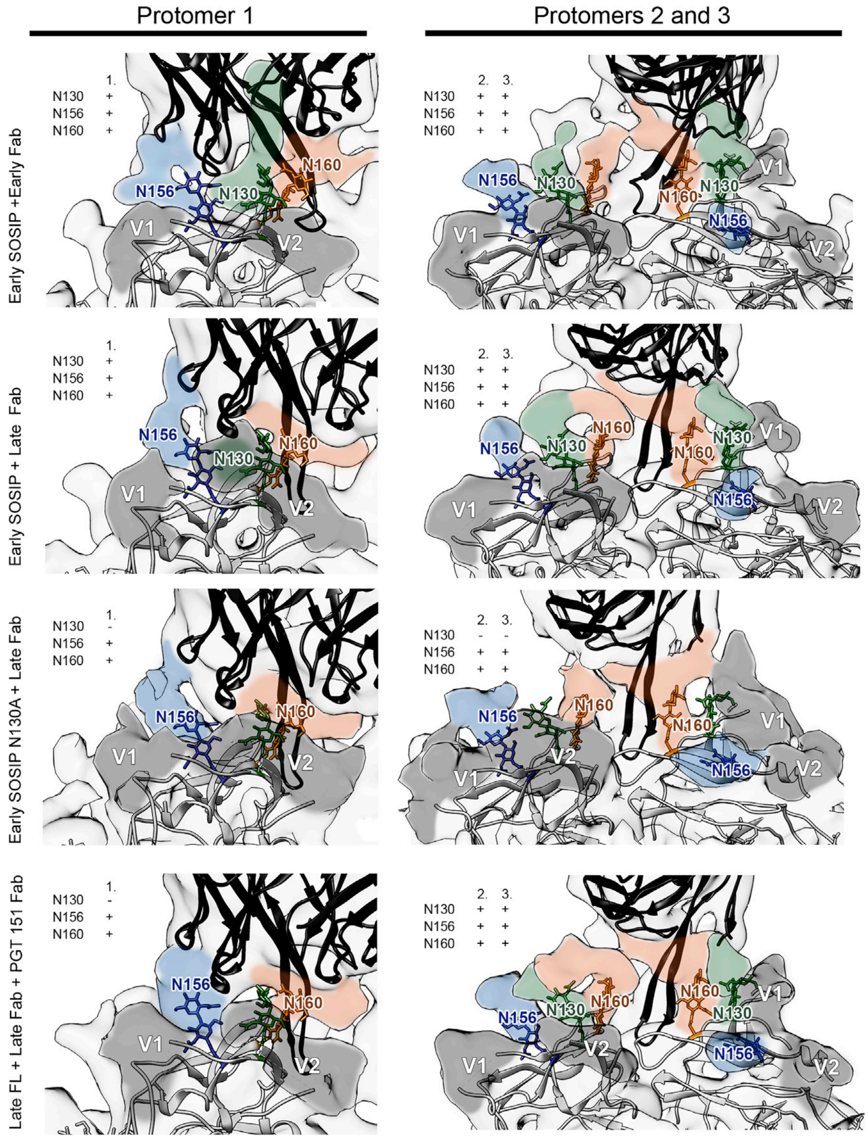

Figure 3. Distribution of Apex Glycans in the Epitopes of Early and Late Env Complexes

Fab crystal structures (black ribbons) and 3.1 Å resolution model of late FL (white ribbons) were docked into cryo-EM maps of autologous complexes and used to estimate the densities attributable to glycans. V1 and V2 loops are highlighted in dark gray. The first 2 or 3 sugars of each glycan are indicated (colored sticks) to define the starting position for each glycan. In overlaid maps, estimated glycan densities are colored blue (N156), green (N130), or orange (N160). Presence or absence of glycan density in each complex is indicated by (+) or (−) in the insets. For clarity, maps were low-pass filtered to 7 Å to match the resolution of early N130A SOSIP-late Fab complex. Glycan N130 shifts out of the binding pocket on the early SOSIP when bound to late Fab versus early Fab.

when comparing the early SOSIP and late FL clones bound to late Fab. However, the N130 glycan appeared to be in direct contact with the early Fab (Figure 3). When the early SOSIP was complexed with the late Fab, we could not observe the N130 contact and the binding angle was identical to late FL complexed with the late Fab. The significance of N130 for the antibody binding angle was assessed further by negative stain EM analysis of early N130A SOSIP-early Fab and by early SOSIP-PCT64-13F, a close relative of early Fab but lacking the CDRL3 SAR motif. In both complexes the Fab bound at an angle identical to early SOSIP-early Fab, confirming that the shallower angle was a property of early Fab and that N130 alone does not define the angle (Figure S2).

The Changing Role of Apex Glycans during Co-evolution

The high-resolution reconstruction of late FL was sufficient to delineate the positions of glycans with atomic detail (Figure S6G). The apical glycans N130, N156, and N160 are within the epitope for the autologous Fabs, with N160 acting as the main glycan contact (Figure 3; Video S3). In protomer 1, the glycan at N156 provides an additional contact, which is a feature not seen with PGT145. In protomers 2 and 3, the glycan at N130 was part of the antibody-contacting glycan canopy, sandwiched between N160 and N156. N130 reaches higher up on the Fab surface compared to other glycans in the canopy, binding to the SAR motif of CDRL3 at the base of CDRH3 (Figure 3). This contacting density was absent in all complexes with late Fab (Figure 3). To confirm the position of N130 in the SOSIP complex and to further study the effect of this glycan, we generated a knockout mutant of early SOSIP (referred as early N130A SOSIP) and solved an 8.2 Å resolution cryo-EM reconstruction of the early N130A SOSIP-late Fab. The missing N130 did not affect the positions of the other glycans in the late Fab liganded interface, except in protomer 3, where the flexible V1 loop was positioned closer to the Fab (Figure 3).

To confirm the role of N130 and to further investigate its importance in co-evolution, we performed neutralization assays on a series of N130 and the adjacent N133 glycan knockout mutants (Figure S3). Early mAb exhibited ~400× greater neutralization (decreased IC_{50}) of early M4 virus and ~100× for late mAb when the N130 glycan was removed. However, late mAb neutralization of late M18 virus was unaffected by the N130-glycan knockout. N133 deletion mutants showed only minor changes in neutralization susceptibility, whereas double mutants reached neutralization levels similar to N130 mutants, confirming that of these two adjacent glycans, neutralization efficiency is mainly dependent on N130. The LMCA behaved similarly to the early

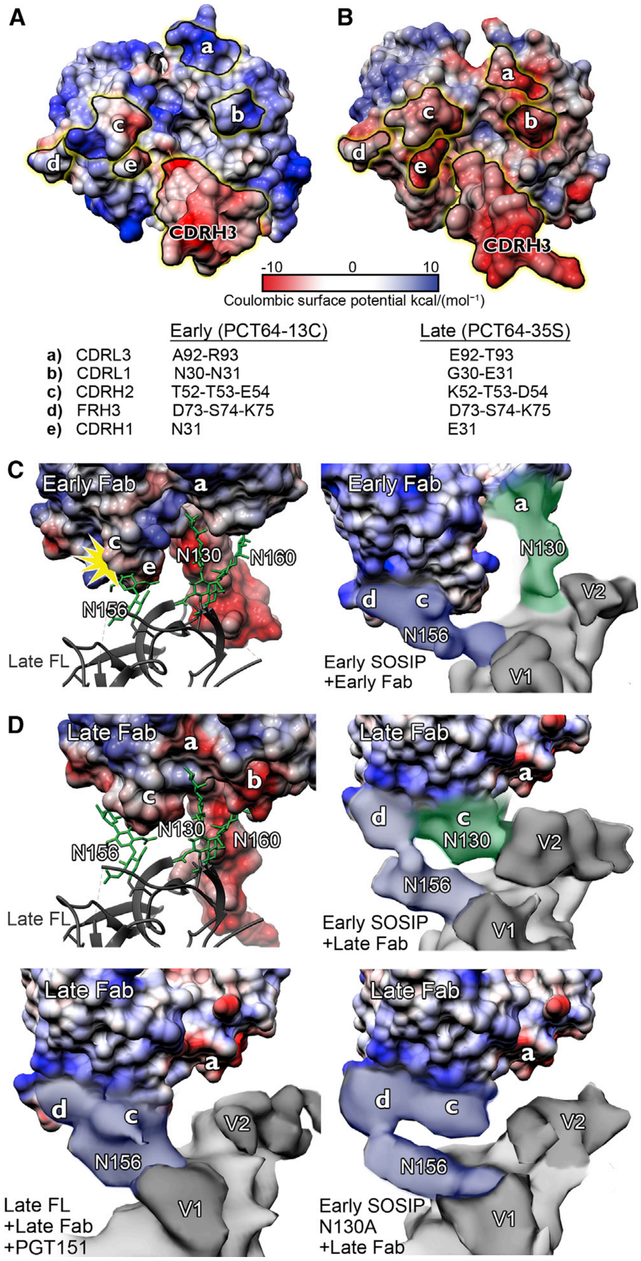

Figure 4. Key Regions Contributing to PCT64 Antibody Surface Charge Switch and Associated Interaction of N130 and N156 at Early and Late Time Point Complexes

(A and B) Electrostatic surface potentials of early Fab (A) and late Fab (B) crystal structures. Patches contributing to the surface charge switch and glycan interactions are indicated.

(C and D) Glycan positions (green sticks) are shown on the 3.1 Å resolution late FL structure (black ribbon) and used as a model for identifying glycan positions in the autologous complex densities (highlighted on overlaid cryo-EM maps).

(C) Early Fab comparison of glycan positioning. (Left) Early Fab is docked into the late FL structure; the angle of approach of the early Fab would create a clash (yellow star) with glycan N156. (Right) Antibody charge patches mapped onto the early SOSIP-early Fab complex map shows N156 contacting patches c and d and N130 contacting patch a.

(D) Late Fab comparison of the glycan positions. (Top left) Late Fab docked into the late FL structure is shown, revealing the potential to accommodate N156. Negative charges are now introduced around the N130 binding pocket (patches a and b). (Top right) In early SOSIP-late Fab complex, N130 contacts patch c. (Lower left) Late FL-late Fab lacking the N130 density similarly to early SOSIP N130A-late Fab complex.

mAb on early M4 virus neutralization in response to N130-glycan knockout, suggesting that N130 participated in the early PC64 Env glycan shielding against the apex-targeting immune response.

The Role of Co-evolution of Surface Charges on Both Binding and Viral Escape

To further investigate the potential N130 glycan binding pocket in the early Fab, electrostatic charge potentials were mapped onto the surfaces of the Fabs, revealing a shift from positive to negative charge near the apparent binding pocket for the glycan (Figures 4 and S4). In the early Fab, the positive charge is mainly contributed by Arg93 of CDRL3 (Figure 4A, patch a). Ala92 and Arg93 are replaced by Glu92 and Thr93 in the late Fab, creating a negatively charged patch (Figure 4B, patch a). In addition, the nearby Asn31 of CDRL1 is mutated to glutamic acid, adding to local negative charge on the surface of the late Fab. By docking the early and late Fabs into our 3.1 Å resolution unliganded model and mapping the antibody charges onto the maps of our autologous complexes, we estimated which surfaces would be contacted by the epitope-forming glycans (Figures 4C and 4D). The core mannose residues of N156 would clash with the early Fab in protomer 1, whereas the glycan is shifted in the complex to accommodate the shallower binding angle (Figure 4C). In the same protomer, N130 is in direct contact with the antibody (patch a in Figure 4C). Complex glycans on gp120 are predominantly terminally sialylated with α2,6-linked sialic acids (Pritchard et al., 2015). In the late Fab, sialic acids of the complex-type N130 glycan would create electrostatically unfavorable interactions with the negatively charged surface of the Fab. This is consistent with the missing N130 glycan contact in the late FL complex cryo-EM map (Figure 4D). Interestingly, the comparison of early and late Fabs revealed evidence of a shift in the N130 contact from CDRL3 to CDRH2 (patches a and c in Figures 4C and 4D), suggesting glycan accommodation. The N156 glycan contact was evident in protomer 1 of all complexes contacting mainly conserved residues D73, S74, and K75 (patch d in Figure 4) and, in the absence of strong N130 contact density, CDRH2 (patch c in Figure 4D). A recent study (Andrabi et al., 2017) suggested that recognition of the sialic acids of the glycan affects antibody maturation and somatically mutated residues were mapped to the same region in CDRH2 that we found to participate in the glycan interaction evolution.

We previously showed that a gradual reduction of positive charge in the V2 loop over the course of infection, particularly at residues 166, 167, and 169, was crucial for virus escape (Landais et al., 2017). The late Env used in our structural studies still contains a net positive charge in V2, representing an intermediate along the path to escape. To examine how this evolving V2 charge manifests itself in the fully folded Env structure, we calculated electrostatic surface potentials for our 3.1 Å resolution late FL structure and for a homology model of the early Env (Figure 5). Residues in the C-strand of the V2 loop identified as crucial for

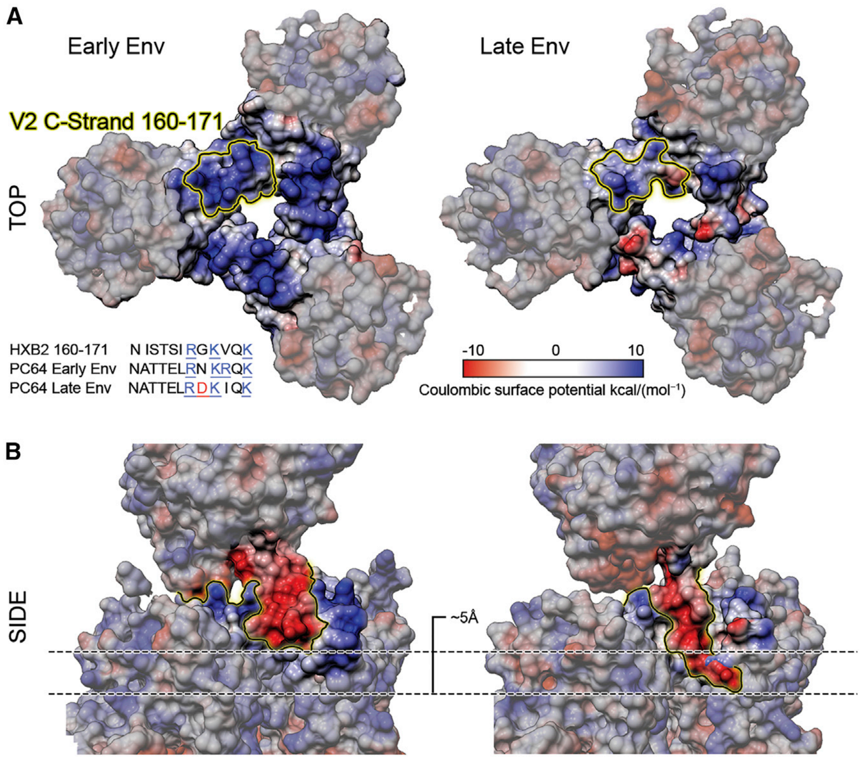

Figure 5. Changes in the PC64 Env Electrostatic Surface Potential between Months 4 and 18

(A) Electrostatic surface potentials shown for top views of the early (homology model) and late (3.1 Å resolution) Env. V2 C-strand sequences are indicated in the inset and highlighted on the apex surface. N168D and R169I contribute to reducing surface charge.

(B) Side views of the same surfaces with early Fab and late Fab cryo-EM maps demonstrating the ~5 Å deeper penetration of the late Fab CDRH3 and of the contact from the negatively charged CDRH3 loop to the positively charged V2 apex in both early and late antibodies.

viral escape form a positively charged ring around the apex cavity. When the PC64 Fab structures were positioned in the cryo-EM maps, the splayed CDRH3 topology of the early antibody would create a larger interacting surface compared to the later time point antibody. Reduction of the superficial positive charge at the apex may in turn drive the antibody to rigidify the CDRH3 tip into a rigid, more extended beta-hairpin that can penetrate deeper into the trimer apex, where it can contact the more conserved positively charged residues (Figures 2A and 5B). Eventually, the V2 C-strand becomes neutral and abrogates the electrostatic attraction between CDRH3 and the apex, thereby likely contributing to viral escape.

Full-Length Env Glycan Shield Processing Is More Uniform Than in SOSIP

We performed a global site-specific mass spectrometry analysis to assess the glycosylation status of all potential N-linked glycosylation sites (PNGS) for both FL and SOSIP constructs (Figure 6). The method provides a semiquantitative assessment of the proportion of the glycosite that contains no glycan, a minimally processed high mannose or hybrid type glycan, or substantially processed complex type glycan. These data revealed several interesting differences in the glycosylation pattern between the soluble and membrane-bound form. In late FL, all glycans except for N133 were >75% either high mannose or complex type glycosylation, indicating a high degree of homogeneity in glycan type at each PNGS. In the late SOSIP version, N130, N133, N141, N187, N197, N234, N301, N409, N616, and N637 were all 25% or more mixed high mannose and complex type or not fully occupied by glycan. Early SOSIP showed a similar level of glycan processing heterogeneity as the late SOSIP, with an additional glycan at N241. Overall, both SOSIP constructs showed similar pattern of glycosite processing. The most significant difference between the early and late SOSIPs was decreased glycosylation at N130 and N133.

In contrast, relative to the late SOSIP, the late FL exhibited a higher degree of processing to complex glycans (e.g., at 130, 186b, 187, 197, 301, 409, and 637; Figure 6). When we grouped glycans based on their location to apex, core, V3/V4/V5, and base to further assess which regions are most affected by the construct type, we saw that the apex and the variable loops are most affected by the construct type (SOSIP versus FL; Figure S5). Of particular interest, the apex glycans of the late FL trimer had 70% complex type glycans compared to ~43% in both early and late SOSIP soluble versions. V3, V4, and V5 loop glycans had 81% complex type in the FL clone compared to 55% complex glycan types in the corresponding regions of SOSIP. Of all the glycosites of the late FL, 72% were occupied by complex glycans, 26% by high mannose glycans, and only 2% of the total was unoccupied. In SOSIP, the respective proportions were 52%, 42%, and 6%. Given that bnAb epitopes, including the PCT64 apex-targeting ones studied here, have significant contacts with glycan residues, often times on variable loops, our analysis of the binding of N130 suggests the potential for favorable interactions with negatively charged sialic acids. This suggests that the antigenicity differences observed could reflect differences in glycan processing in SOSIP and FL clones, e.g., for PC64 at N130. Such differences could also account for our inability to observe binding of early SOSIP to late Fab.

DISCUSSION

Soluble SOSIP Env trimers offer a highly attractive platform for immunogen design and vaccine development (Sanders and Moore, 2017). Here, we set out to compare wild-type, FL Env with corresponding soluble SOSIP immunogens and investigate the co-evolutionary mechanisms between Env and an apex-targeting antibody lineage to help guide immunogen design.

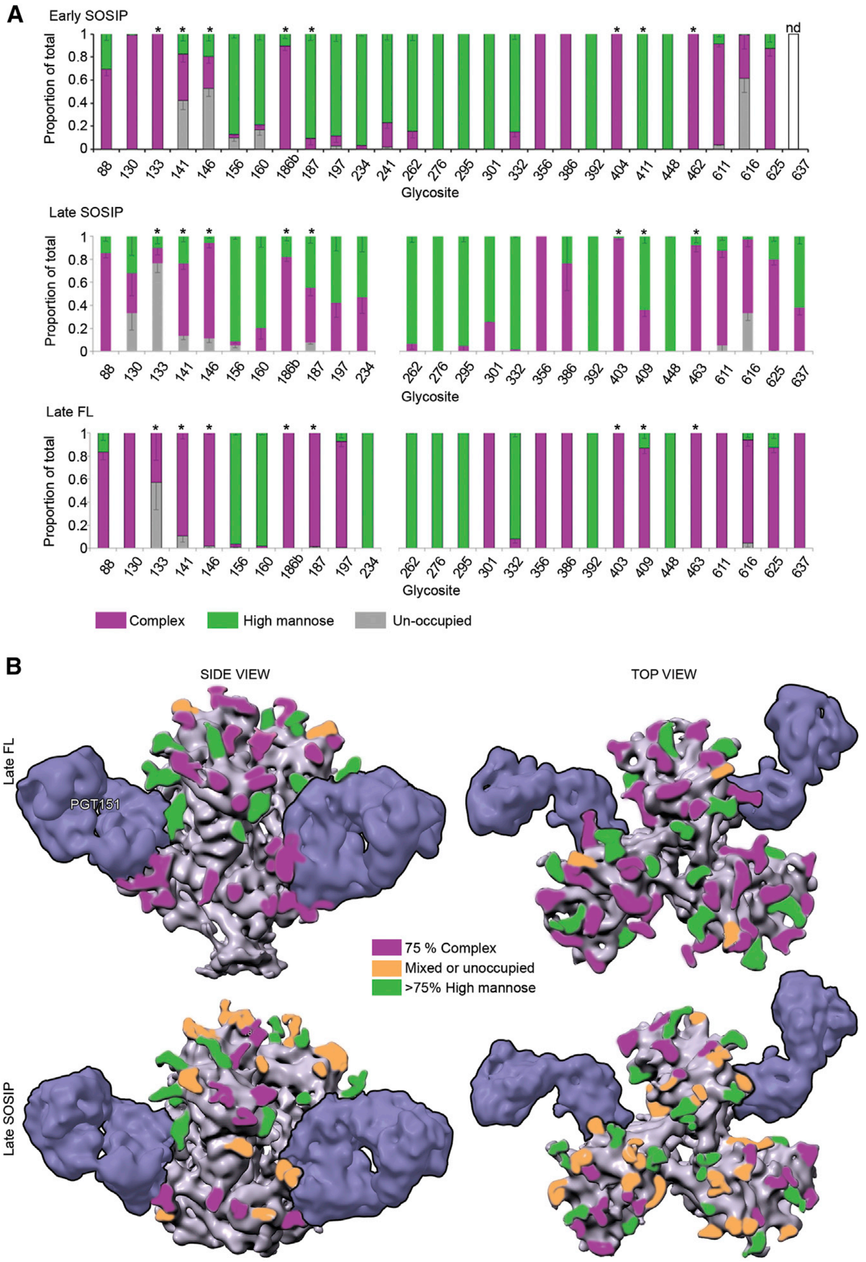

Figure 6. Global Site-Specific N-Glycosylation Analysis of Early SOSIP, Late SOSIP, and Late Full-Length Env

(A) N-glycan analysis on various PC64 Env constructs. Proportions of unoccupied sites and sites occupied by either high mannose or complex glycans are shown. Stars above bars indicate glycans that were undetectable in cryo-EM maps due to structural flexibility.

(B) Mapping of glycosylation differences between SOSIP and FL Env on the surface of corresponding cryo-EM density maps with two PGT151 Fabs bound per trimer.

Consistent with previous observations, the SOSIP trimer structure is an accurate mimic of the native, FL trimer. Whereas SOSIP trimer glycosylation patterns are overall similar, we observed site-specific glycosylation differences between soluble immunogens and native, membrane-embedded Env. These differences may be an important consideration for epitope-focused vaccine approaches. Differences in glycosylation of soluble SOSIP constructs have also been observed in earlier mass spectrometric studies, and the enzymatic glycosylation processing in the ER and Golgi can differentially impact soluble and transmembrane proteins (Behrens et al., 2016; Cao et al., 2017; Go et al., 2015). Although, as discussed below, if the apex antibody response can be focused on N160 glycans alone, this may not be a problem, as N160 is consistently high mannose in both SOSIP and FL Env.

In the PCT64 lineage, we observe an ~20° change in the binding angle and concomitant rigidification of CDRH3 during the course of antibody evolution. The early precursors of PCT64, therefore, likely utilize a loosely ordered, negatively charged CDRH3 to bind the positively charged Env apex. Thus, the early angle of approach is not fixed but becomes more vertical with respect to the Env trimer three-fold axis and then becomes fixed during somatic hypermutation and evolution against Env during infection. Evolution of the binding angle appears to occur simultaneously with deeper penetration of CDRH3 into the Env apex and modulation of the antibody surface charge to accommodate the glycan shield. Additional structural analysis of intermediate complexes, e.g., at time points 12 and 24 months post-infection, in combination with mutagenesis studies will be necessary to fully understand the roles of Ab and Env mutations in the divergence points of antibody evolution and acquisition of breadth.

Whereas the best PCT64 monoclonal antibody isolated has several attractive properties, it only has limited breadth, peaking at 29% neutralization efficiency against a cross-clade panel at 35 months post-infection, and the autologous virus escapes by month 18 (Landais et al., 2017). Thus, the co-evolution that occurred in donor PC64 may have led this antibody lineage ultimately down a path that limited its breadth and potency (Figure 7). The shallower approach angle in the early antibody becomes steeper over time but ultimately is fixed at an angle that makes the antibody susceptible to changes in V1 and V2 length and glycosylation. PGT145, on the other hand, has a more vertical angle of approach around the trimer 3-fold axis that enables it to avoid V1 and V2, contacting mainly the conserved N160

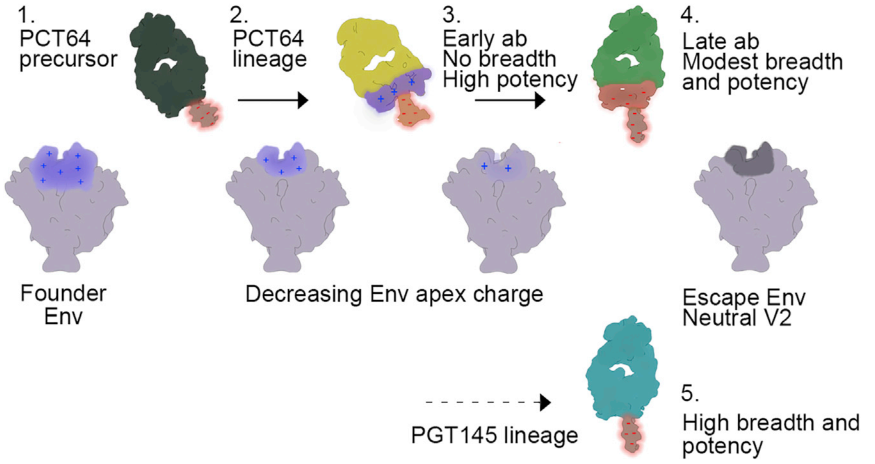

Figure 7. Co-evolution Mechanisms of the PC64 Virus and Immune System and Lessons for Immunogen Design

The founder PC64 virus has a strong electropositive charge at the Env three-fold axis. (1) PCT64 antibody precursor with anionic CDRH3 targets the apex and initiates (2) the lineage maturation. Interplay between the virus and immune response results in a gradual decrease of the positive charge of the V2 apex plus changes in the glycan composition of Env and (3) the antibody evolving a steeper angle of approach, rigid CDRH3 allowing deeper penetration into the three-fold axis, and modulation of the charge of the surface contacting the glycan canopy. These changes lead to high potency against autologous virus but no breadth against heterologous viruses. (4) Further maturation leads to modest breadth and potency. At later stages of infection, these changes limit the antibody lineage evolution while the apex charge becomes neutral, leading to virus escape. PC64 embodies several of the properties of the (5) broad and potent PGT145 bnAb lineage but does differentiate down a path that prevents further productive maturation. A potential path of PC64 response may be diverted toward PGT145-like response by altering V1 and V2 loop lengths and by adding or removing apex glycans.

glycans, resulting in the broadest and most potent class of apex bnAbs. Whereas PGT145 family of bnAbs are not the most desirable targets due to the extra-long CDRH3 (33 aas) that requires a rare insertion (Walker et al., 2011), the PCT64 lineage represents a potential path to the same class with a shorter CDRH3 (25 aas).

Current methods in rational, structure-based vaccine design are centered on recapitulating known bnAb lineages to ultimately elicit a known bnAb family from chronic infection. It has been elegantly shown that such an approach may be possible for the VRC01 class of antibodies that target the CD4 binding site (Jardine et al., 2013, 2016) as well as the PGT121 class of antibodies that target the N332 supersite (Escolano et al., 2016; Steichen et al., 2016). These approaches are predicated on a fixed angle of approach from germline and subsequent somatic hypermutation (SHM) to improve affinity and navigate through the surrounding glycans. Germline-targeting immunogens and rational boosts intended to recapitulate the infection model have therefore been derived via a combination of rational design and mammalian display using the known bnAbs. Thus, the immunogens are not surprisingly biased toward the bnAbs used in the "training" process. Here, we propose a somewhat different approach, one that is Env focused, rather than targeting interaction with specific bnAbs or a particular maturation pathway. Our data suggest that, for apex-directed responses, it is desirable to stimulate germline Abs and/or early intermediates with long, flexible, and anionic CDRH3 that can bind around the three-fold axis at the apex using a germline-targeting immunogen. It is presumably desirable to enrich as many germline B cell precursors that have this property in an epitope-focused manner rather than a germline-specific manner. PCT64, as we describe above, is one such germline that we think has great potential despite not achieving high breadth in the PC64 donor (Figure 7), but we also hypothesize there will be other germline precursors. Boosting immunogens could then be designed to influence the angle of approach using strategies similar to those seen in PCT64 lineage, which we consider a pluripotent intermediate before CDRH3 stabilization. CDRH3 may be accomplished by adding glycans to the apex that prevent the non-vertical binding angle relative to the apex exhibited by PG9/16, CAP256, or PCT64. The ability to approach Env at a near vertical angle and target only the highly conserved oligomannose N160 glycans in both FL and SOSIP Env and the underlying cationic trimer apex is a hallmark of breadth and potency in PGT145/PGDM1400. The PCT64 lineage achieves the latter, but not the former, as its angle of approach makes it susceptible to V1 and V2 loop length and nearby glycans. Hence, we propose that we should not attempt to reverse engineer the mature PCT64 lineage but rather divert the PCT64 lineage early on to obtain increased breadth and potency. A boosting immunogen that contains a long and stable V1 and particularly V2 may drive this outcome by selecting only CDRH3 variants that adopt an appropriate conformation such that the antibody approaches at an angle orthogonal to the apex. This type of approach attempts to induce apex epitope-focused bnAbs with the correct properties but without attempting to select for specific mutations.

EXPERIMENTAL PROCEDURES

Full-Length Env Expression and Purification

Details of the expression screening are in Supplemental Information and in Figure S1. Large-scale expression and purification was performed as follows: unmodified Env sequences of PC64 lineage clones were inserted into pcDNA3.1. 8 L of HEK293F cells were transfected with 250 μg of Env DNA per liter of cells and co-transfected with 62.5 μg furin DNA per liter. Cells were transfected at ~1.8 million cells/mL and harvested 3 days after transfection. FL Env was purified following methods previously described by Blattner et al. (2014) and Lee et al. (2016) with slight modifications. Briefly, PGT151 with a tobacco etch virus (TEV) cleavage site inserted between the Fab and Fc regions was added at 100 μg/mL of PBS washed and resuspended intact cells. 0.5 mL Protein A slurry was used per liter of cells. All buffers were as in Lee et al. (2016), except that L-cysteine was omitted from wash buffer 3.

SOSIP Expression and Purification

SOSIP versions of PC64 Env were expressed and purified as described previously (Sanders et al., 2013). Following 6-day transient expression in HEK293F cells, trimers were extracted and purified using lectin affinity chromatography

or 2G12 antibody-based affinity chromatography followed by size-exclusion chromatography.

Fab Expression and Purification

The Fab fragments of LMCA-CDRL3$_{SAR}$, PCT64-13C (early Fab), and PCT64-35S (late Fab) were expressed in FreeStyle 293F cells (Invitrogen), with a heavy chain:light chain ratio of 2:1 and co-transfection with protein-tyrosine sulfotransferase 1 (TPST1) to enhance tyrosine sulfation. Fab fragments were subsequently purified by affinity chromatography (anti-human kappa) and cation exchange chromatography (Mono S 10/100 GL). Purified fractions were analyzed by gel electrophoresis and exchanged into 20 mM sodium acetate (pH 5.6; LMCA-CDRL3$_{SAR}$) and 20 mM Tris and 150 mM NaCl (pH 7.4; early and late Fab), respectively. The proteins were subjected to crystallization trials with the IAVI/JCSG/TSRI CrystalMation robotic system (Rigaku) at either 20°C (LMCA-CDRL3$_{SAR}$) or both 4°C and 20°C (early and late Fab).

Fab Crystallization, Data Collection, Structure Determination, and Refinement

Crystals of late Fab at 8 mg/mL were obtained at 20°C from a condition containing 0.17 M sodium acetate, 0.085 M Tris (pH 8.5), 15% (v/v) glycerol and 25.5% (w/v) polyethylene glycol (PEG) 4000. Early Fab at 6.7 mg/mL crystallized in 10% (v/v) 2-propanol, 10% (v/v) glycerol, 0.1 M HEPES (pH 7.5), and 20% PEG 2000 (w/v). LMCA-CDRL3$_{SAR}$ crystallized at 11.9 mg/mL in 0.1 M Tris (pH 8), 1 M LiCl, and 10% PEG 6000. Crystals were cryo-cooled by immediate plunging into liquid nitrogen, with the LMCA-CDRL3$_{SAR}$ crystal cryo-protected in 70% well solution and 30% glycerol. Data for early and late Fabs were collected at the APS GM/CA Structural Biology facility on a Pilatus3 6M detector at beamline ID-D23. Data for the LMCA-CDRL3$_{SAR}$ were collected at the SSRL 12-2 beamline, on a Dectris Pilatus3 6M detector. Data were processed using HKL-2000 (Otwinowski and Minor, 1997). Molecular replacement was carried out using Phaser (McCoy et al., 2007), with a loop-truncated version of PDB ID: 5FEH (PCT64-35B), divided into variable and constant regions, as the initial search model. Model building and refinement were conducted in COOT (Emsley et al., 2010) and PHENIX (Afonine et al., 2012), respectively. PyMOL was used for the rendering of images (Schrödinger, LLC, 2015), and the structures were validated with MolProbity (Chen et al., 2010).

EM Sample Preparation

For the late FL sample, 5 μL of Env at 7 mg/mL was mixed with or without 2.8 μL of the MPER-targeting Fab DH511 at 8.5 mg/mL, 1 μL of 1 mM lipid mix (1:1 dioleoyl-phosphocholine [DOPC]:cholesterol hemissucinate [CHS]), and 2.2 μL of gel filtration buffer in a total reaction volume of 8.2 μL or 11 μL. Detergent was removed at 4°C by four consecutive additions of 3–5 biobeads (Bio-Rad) at 1-hr intervals. After the last incubation, 1 μL of 0.01% (w/v) A8-35 amphiphol was mixed with 3 μL of sample and frozen on plasma cleaned 2/2 C-Flat Holey Carbon grids (Protochips) using the Vitrobot mark IV set to 6 s blot time, blot force 0, and 10 s wait time. Neither the MPER-targeting antibody nor the lipid mixture showed an effect on the structure of the Env TM or CT part of the complex. The highest resolution reconstruction was obtained from an MPER Fab-containing sample, but due to poor Fab occupancy and heterogeneity, MPER, TM, and CT regions were masked out during reconstruction to improve the resolution of the ectodomain. Late FL-late Fab was prepared as follows: 175 μg of Env was mixed with 150 μg of late Fab and incubated overnight at 4°C followed by size exclusion chromatography for a final complex concentration (0.9 mg/mL). Samples were diluted 1:10 in TBS and then 3 μL were frozen on 1.2/1.3 μm hole size Quantifoil grids overlaid with a thin carbon film that was deposited manually on the grid. 3 μL of diluted sample was used per grid, blotted off, and plunge frozen using a manual plunger. All SOSIP cryo-EM samples were complexed prior to size exclusion purification with a 1:3 Env:Fab molar ratio for the apex-targeting Fabs or 1:6 for PGT151 Fab. The purified final sample was concentrated to 5 or 6 mg/mL. 3 μL of sample were mixed with 1 μL of 1.8 mM dodecyl maltoside (DDM) and frozen on 2/2 C-Flat grids using the Vitrobot mark IV (Thermo Fisher Scientific) with 5 or 6 s blot time, blot force 0, and wait time of 10 s. Negative stain samples were prepared as follows: 3 μL of purified trimer was applied to plasma-cleaned 400 mesh Cu grids (Ted Pella) at ~0.04 mg/mL, blotted off, and followed by two rounds of staining with 3 μL 2% (w/v) uranyl formate.

EM Data Collection

A summary of imaging conditions is presented in Table S1. Late FL, early SOSIP with early or late Fab, early N130A SOSIP with late Fab, and late SOSIP with PGT151 Fab were imaged using a Titan Krios (Thermo Fisher Scientific) operating at 300 keV equipped with a K2 Summit direct electron detector (Gatan). Late FL with late Fab was imaged using a Talos Arctica (Thermo Fisher Scientific) operating at 200 keV and a K2 direct electron detector (Gatan). Negative stain data were collected with Tecnai Spirit (Thermo Fisher Scientific). All data were collected using the Leginon automated image acquisition software (Potter et al., 1999).

EM Data Processing

Details of the software and general workflow are presented in Supplemental Information. In short, movie micrographs were aligned and dose weighted using MotionCor2 (Zheng et al., 2017), and contrast transfer functions (CTFs) were calculated for each micrograph using GCTF (Zhang, 2016). Particles were picked from aligned images using Relion template picking (Scheres, 2015) or DoGPicker (Voss et al., 2009). After these pre-processing steps, all subsequent downstream single-particle operations were performed with gpu-accelerated Relion/2.0 (Kimanius et al., 2016). Resolutions were estimated by Fourier shell correlation of independently refined half maps (Fourier shell correlation [FSC] = 0.143 for cryo; 0.5 for negative stain; Figure S7).

Model Building and Figure Preparation

Model building for late FL in complex with PGT151 was initiated by preparing a homology model with SWISS-MODEL (Arnold et al., 2006) using JRFLΔCT (PDB ID: 5FUU) as a template. Separate gp120 and gp41 models were generated, fitted into the cryo-EM map, and combined as a trimeric complex with two copies of PGT151 Fabs using UCSF Chimera (Pettersen et al., 2004). Initial refinement was done using Rosetta density-guided local refinement (DiMaio et al., 2015). Glycans were added as idealized Man_9 models and trimmed to match EM map densities. On the PGT151 Fab contacting interfaces where glycans were stabilized, N637, N616, N611, and N262 glycans were built based on the glycoforms present on JRFLΔCT (PDB ID: 5FUU). Final refinements were done iteratively with Phenix real-space refinement (Adams et al., 2010) and manual building using COOT (Emsley et al., 2010). Model validation was performed using MolProbity (Chen et al., 2010) and EMRinger (Barad et al., 2015; Table S2). Electrostatic potential of the surfaces was calculated and colored using the “calculate Coulombic surface” function in Chimera (Pettersen et al., 2004). All figures were prepared using UCSF Chimera and ChimeraX (Goddard et al., 2018; Pettersen et al., 2004).

Global Site-Specific N-Glycosylation Analysis

Envs were digested and deglycosylated as previously described (Cao et al., 2017). Briefly, approximately 30 μg of Env was denatured with 8 M urea in 0.1 M ammonium acetate (pH 6), followed by DTT and iodoacetamide treatments to reduce and alkylate the protein. The resulting protein was divided into five aliquots for the proteolytic digestions, including triple digestion (Gatlin et al., 2000), chymotrypsin, and the combination of trypsin and chymotrypsin. The protease enzymes were then denatured at 100°C for 5 min. Each of the samples generated from different proteolytic digestions was deglycosylated with Endo H, followed by PNGase F treatment. The samples were then analyzed on a Fusion Orbitrap tribrid mass spectrometer (Thermo Fisher Scientific). MS/MS spectra were searched against the European Bioinformatic Institute (IPI) *Bos taurus* protein database, including the sequences of HIV-1 Env trimers analyzed in this study using the Integrated Proteomics Pipeline Ver. 5.1.2 (Eng et al., 1994; Tabb et al., 2002; Xu et al., 2015). Each peak was smoothed and fitted to Gaussian distribution to calculate the relative abundance of peptide using peak area.

DATA AND SOFTWARE AVAILABILITY

The accession numbers for the following structures reported in this paper are (PCT64-LMCA [CDRL3$_{SAR}$]) PDB: 6CA9, (PCT64-13C [early Fab]) PDB: 6CA7, (PCT64-35S [late Fab]) PDB: 6CA6, and (PC64M18C043 FL [late FL] + PGT151

Fab) PDB: 6DCQ. The accession numbers for the following EM density maps reported in this paper are (PC64M18C043 FL [late FL] + PGT151 Fab) EMDB: EMD-7858, (PC64M18C043 FL [late FL] + PGT151 Fab + PCT64-35S [late Fab]) EMDB: EMD-7859, (PC64M18C043 SOSIP [late SOSIP] + PGT151 Fab) EMDB: EMD-7860, (PC64M4C054 SOSIP [early SOSIP] + PCT-13C Fab [early Fab]) EMDB: EMD-7863, (PC64M4C054 SOSIP [early SOSIP] + PCT-35S Fab [late Fab]) EMDB: EMD-7865, (PC64M4C054 N130A SOSIP [early N130A SOSIP] + PCT-35S Fab [late Fab]) EMDB: EMD-7866, (PC64M4C054 [early FL] + PGT151 Fab + PCT-13C Fab [early Fab]) EMDB: EMD-7861, (PC64M4C054 N130A SOSIP [early N130A SOSIP] + PCT-13C Fab [early Fab]) EMDB: EMD-7864, and (PC64M4C054 [early SOSIP] +PCT-13F Fab) EMDB: EMD-7862.

SUPPLEMENTAL INFORMATION

Supplemental Information includes Supplemental Experimental Procedures, seven figures, two tables, and three videos and can be found with this article online at https://doi.org/10.1016/j.celrep.2018.05.046.

ACKNOWLEDGMENTS

We thank Hannah Turner, Travis Nieusma, and Bill Anderson for assistance with microscope management, IAVI Protocol C and associated investigators for providing access to sample collection, and Gabe Ozorowski and Lauren Holden for critical reading of the manuscript. This work was supported by NIH grant UM1 AI100663 (Scripps CHAVI-ID), the International AIDS Vaccine Initiative Neutralizing Antibody Center, and the Collaboration for AIDS Vaccine Discovery OPP1115782 and OPP1084519 (Bill and Melinda Gates Foundation). This work was partially funded by IAVI with the generous support of USAID, the Ministry of Foreign Affairs of the Netherlands, and the Bill and Melinda Gates Foundation; a full list of IAVI donors is available at http://www.iavi.org. The contents of this manuscript are the responsibility of the authors and do not necessarily reflect the views of USAID or the US Government. Use of the Stanford Synchrotron Radiation Lightsource, SLAC National Accelerator Laboratory, is supported by the US Department of Energy, Office of Science, Office of Basic Energy Sciences under contract no. DE-AC02-76SF00515. The SSRL Structural Molecular Biology Program is supported by the DOE Office of Biological and Environmental Research and by the NIH, National Institute of General Medical Sciences (including P41GM103393). The contents of this publication are solely the responsibility of the authors and do not necessarily represent the official views of NIGMS or the NIH. This research also used resources of the Advanced Photon Source, a US Department of Energy (DOE) Office of Science User Facility operated for the DOE Office of Science by Argonne National Laboratory under contract no. DE-AC02-06CH11357.

AUTHOR CONTRIBUTIONS

Project Coordination, K.R.; Experiment Design, K.R., Z.T.B., S.M., L.C., E.L., and A.B.W.; Sample Purification and EM Sample Preparation, K.R., M.W., J.L.T., J.C., and J.U.; EM Imaging, K.R. and Z.T.B.; Data Processing, Z.T.B.; Crystal Studies, S.M. and O.O.; Model Building, K.R., S.M., and O.O.; Glycosylation Analysis, L.C.; Env Clone and PC Cohort Sample Coordination and Neutralization Assays, E.L.; Manuscript Writing, K.R., P.P., J.C.P., I.A.W., and A.B.W. All authors were asked to comment on the manuscript.

DECLARATION OF INTERESTS

The authors declare no competing interests.

Received: February 20, 2018
Revised: April 9, 2018
Accepted: May 14, 2018
Published: June 12, 2018

REFERENCES

Adams, P.D., Afonine, P.V., Bunkóczi, G., Chen, V.B., Davis, I.W., Echols, N., Headd, J.J., Hung, L.-W., Kapral, G.J., Grosse-Kunstleve, R.W., et al. (2010). PHENIX: a comprehensive Python-based system for macromolecular structure solution. Acta Crystallogr. D Biol. Crystallogr. *66*, 213–221.

Afonine, P.V., Grosse-Kunstleve, R.W., Echols, N., Headd, J.J., Moriarty, N.W., Mustyakimov, M., Terwilliger, T.C., Urzhumtsev, A., Zwart, P.H., and Adams, P.D. (2012). Towards automated crystallographic structure refinement with phenix.refine. Acta Crystallogr. D Biol. Crystallogr. *68*, 352–367.

Andrabi, R., Su, C.-Y., Liang, C.-H., Shivatare, S.S., Briney, B., Voss, J.E., Nawazi, S.K., Wu, C.-Y., Wong, C.-H., and Burton, D.R. (2017). Glycans function as anchors for antibodies and help drive HIV broadly neutralizing antibody development. Immunity *47*, 1004.

Arnold, K., Bordoli, L., Kopp, J., and Schwede, T. (2006). The SWISS-MODEL workspace: a web-based environment for protein structure homology modelling. Bioinformatics *22*, 195–201.

Barad, B.A., Echols, N., Wang, R.Y.-R., Cheng, Y., DiMaio, F., Adams, P.D., and Fraser, J.S. (2015). EMRinger: side chain-directed model and map validation for 3D cryo-electron microscopy. Nat. Methods *12*, 943–946.

Behrens, A.-J., Vasiljevic, S., Pritchard, L.K., Harvey, D.J., Andev, R.S., Krumm, S.A., Struwe, W.B., Cupo, A., Kumar, A., Zitzmann, N., et al. (2016). Composition and antigenic effects of individual glycan sites of a trimeric HIV-1 envelope glycoprotein. Cell Rep. *14*, 2695–2706.

Blattner, C., Lee, J.H., Sliepen, K., Derking, R., Falkowska, E., de la Peña, A.T., Cupo, A., Julien, J.P., van Gils, M., Lee, P.S., et al. (2014). Structural delineation of a quaternary, cleavage-dependent epitope at the gp41-gp120 interface on intact HIV-1 Env trimers. Immunity *40*, 669–680.

Bonsignori, M., Zhou, T., Sheng, Z., Chen, L., Gao, F., Joyce, M.G., Ozorowski, G., Chuang, G.-Y., Schramm, C.A., Wiehe, K., et al.; NISC Comparative Sequencing Program (2016). Maturation pathway from germline to broad HIV-1 neutralizer of a CD4-mimic antibody. Cell *165*, 449–463.

Briney, B., Sok, D., Jardine, J.G., Kulp, D.W., Skog, P., Menis, S., Jacak, R., Kalyuzhniy, O., de Val, N., Sesterhenn, F., et al. (2016). Tailored immunogens direct affinity maturation toward HIV neutralizing antibodies. Cell *166*, 1459–1470.e11.

Burton, D.R., and Hangartner, L. (2016). Broadly neutralizing antibodies to HIV and their role in vaccine design. Annu. Rev. Immunol. *34*, 635–659.

Cao, L., Diedrich, J.K., Kulp, D.W., Pauthner, M., He, L., Park, S.R., Sok, D., Su, C.-Y., Delahunty, C.M., Menis, S., et al. (2017). Global site-specific N-glycosylation analysis of HIV envelope glycoprotein. Nat. Commun. *8*, 14954.

Chen, V.B., Arendall, W.B., 3rd, Headd, J.J., Keedy, D.A., Immormino, R.M., Kapral, G.J., Murray, L.W., Richardson, J.S., and Richardson, D.C. (2010). MolProbity: all-atom structure validation for macromolecular crystallography. Acta Crystallogr. D Biol. Crystallogr. *66*, 12–21.

Cheng, C., Pancera, M., Bossert, A., Schmidt, S.D., Chen, R.E., Chen, X., Druz, A., Narpala, S., Doria-Rose, N.A., McDermott, A.B., et al. (2015). Immunogenicity of a prefusion HIV-1 envelope trimer in complex with a quaternary-structure-specific antibody. J. Virol. *90*, 2740–2755.

DiMaio, F., Song, Y., Li, X., Brunner, M.J., Xu, C., Conticello, V., Egelman, E., Marlovits, T., Cheng, Y., and Baker, D. (2015). Atomic-accuracy models from 4.5-Å cryo-electron microscopy data with density-guided iterative local refinement. Nat. Methods *12*, 361–365.

Doria-Rose, N.A., Bhiman, J.N., Roark, R.S., Schramm, C.A., Gorman, J., Chuang, G.-Y., Pancera, M., Cale, E.M., Ernandes, M.J., Louder, M.K., et al. (2015). New member of the V1V2-directed CAP256-VRC26 lineage that shows increased breadth and exceptional potency. J. Virol. *90*, 76–91.

Dubrovskaya, V., Guenaga, J., de Val, N., Wilson, R., Feng, Y., Movsesyan, A., Karlsson Hedestam, G.B., Ward, A.B., and Wyatt, R.T. (2017). Targeted N-glycan deletion at the receptor-binding site retains HIV Env NFL trimer integrity and accelerates the elicited antibody response. PLoS Pathog. *13*, e1006614.

Emsley, P., Lohkamp, B., Scott, W.G., and Cowtan, K. (2010). Features and development of Coot. Acta Crystallogr. D Biol. Crystallogr. *66*, 486–501.

Eng, J.K., McCormack, A.L., and Yates, J.R. (1994). An approach to correlate tandem mass spectral data of peptides with amino acid sequences in a protein database. J. Am. Soc. Mass Spectrom. *5*, 976–989.

Escolano, A., Steichen, J.M., Dosenovic, P., Kulp, D.W., Golijanin, J., Sok, D., Freund, N.T., Gitlin, A.D., Oliveira, T., Araki, T., et al. (2016). Sequential immunization elicits broadly neutralizing anti-HIV-1 antibodies in Ig knockin mice. Cell *166*, 1445–1458.e12.

Feng, Y., Tran, K., Bale, S., Kumar, S., Guenaga, J., Wilson, R., de Val, N., Arendt, H., DeStefano, J., Ward, A.B., and Wyatt, R.T. (2016). Thermostability of well-ordered HIV spikes correlates with the elicitation of autologous tier 2 neutralizing antibodies. PLoS Pathog. *12*, e1005767.

Garces, F., Sok, D., Kong, L., McBride, R., Kim, H.J., Saye-Francisco, K.F., Julien, J.-P., Hua, Y., Cupo, A., Moore, J.P., et al. (2014). Structural evolution of glycan recognition by a family of potent HIV antibodies. Cell *159*, 69–79.

Gatlin, C.L., Eng, J.K., Cross, S.T., Detter, J.C., and Yates, J.R., 3rd. (2000). Automated identification of amino acid sequence variations in proteins by HPLC/microspray tandem mass spectrometry. Anal. Chem. *72*, 757–763.

Go, E.P., Cupo, A., Ringe, R., Pugach, P., Moore, J.P., and Desaire, H. (2015). Native conformation and canonical disulfide bond formation are interlinked properties of HIV-1 Env glycoproteins. J. Virol. *90*, 2884–2894.

Goddard, T.D., Huang, C.C., Meng, E.C., Pettersen, E.F., Couch, G.S., Morris, J.H., and Ferrin, T.E. (2018). UCSF ChimeraX: Meeting modern challenges in visualization and analysis. Protein Sci. *27*, 14–25.

Jardine, J., Julien, J.-P., Menis, S., Ota, T., Kalyuzhniy, O., McGuire, A., Sok, D., Huang, P.-S., MacPherson, S., Jones, M., et al. (2013). Rational HIV immunogen design to target specific germline B cell receptors. Science *340*, 711–716.

Jardine, J.G., Kulp, D.W., Havenar-Daughton, C., Sarkar, A., Briney, B., Sok, D., Sesterhenn, F., Ereño-Orbea, J., Kalyuzhniy, O., Deresa, I., et al. (2016). HIV-1 broadly neutralizing antibody precursor B cells revealed by germline-targeting immunogen. Science *351*, 1458–1463.

Johnson, W.E., and Desrosiers, R.C. (2002). Viral persistence: HIV's strategies of immune system evasion. Annu. Rev. Med. *53*, 499–518.

Kimanius, D., Forsberg, B.O., Scheres, S.H., and Lindahl, E. (2016). Accelerated cryo-EM structure determination with parallelisation using GPUs in RELION-2. eLife *5*, 19.

Klasse, P.J., LaBranche, C.C., Ketas, T.J., Ozorowski, G., Cupo, A., Pugach, P., Ringe, R.P., Golabek, M., van Gils, M.J., Guttman, M., et al. (2016). Sequential and simultaneous immunization of rabbits with HIV-1 envelope glycoprotein SOSIP.664 trimers from clades A, B and C. PLoS Pathog. *12*, e1005864.

Landais, E., Huang, X., Havenar-Daughton, C., Murrell, B., Price, M.A., Wickramasinghe, L., Ramos, A., Bian, C.B., Simek, M., Allen, S., et al. (2016). Broadly neutralizing antibody responses in a large longitudinal sub-Saharan HIV primary infection cohort. PLoS Pathog. *12*, e1005369.

Landais, E., Murrell, B., Briney, B., Murrell, S., Rantalainen, K., Berndsen, Z.T., Ramos, A., Wickramasinghe, L., Smith, M.L., Eren, K., et al.; IAVI Protocol C Investigators; IAVI African HIV Research Network (2017). HIV envelope glycoform heterogeneity and localized diversity govern the initiation and maturation of a V2 apex broadly neutralizing antibody lineage. Immunity *47*, 990–1003.e9.

Lee, J.H., Ozorowski, G., and Ward, A.B. (2016). Cryo-EM structure of a native, fully glycosylated, cleaved HIV-1 envelope trimer. Science *351*, 1043–1048.

Lee, J.H., Andrabi, R., Su, C.-Y., Yasmeen, A., Julien, J.-P., Kong, L., Wu, N.C., McBride, R., Sok, D., Pauthner, M., et al. (2017). A broadly neutralizing antibody targets the dynamic HIV envelope trimer apex via a long, rigidified, and anionic β-hairpin structure. Immunity *46*, 690–702.

MacLeod, D.T., Choi, N.M., Briney, B., Garces, F., Ver, L.S., Landais, E., Murrell, B., Wrin, T., Kilembe, W., Liang, C.H., et al.; IAVI Protocol C Investigators & The IAVI African HIV Research Network (2016). Early antibody lineage diversification and independent limb maturation lead to broad HIV-1 neutralization targeting the Env high-mannose patch. Immunity *44*, 1215–1226.

McCoy, A.J., Grosse-Kunstleve, R.W., Adams, P.D., Winn, M.D., Storoni, L.C., and Read, R.J. (2007). Phaser crystallographic software. J. Appl. Cryst. *40*, 658–674.

McCoy, L.E., van Gils, M.J., Ozorowski, G., Messmer, T., Briney, B., Voss, J.E., Kulp, D.W., Macauley, M.S., Sok, D., Pauthner, M., et al. (2016). Holes in the glycan shield of the native HIV envelope are a target of trimer-elicited neutralizing antibodies. Cell Rep. *16*, 2327–2338.

McLellan, J.S., Pancera, M., Carrico, C., Gorman, J., Julien, J.-P., Khayat, R., Louder, R., Pejchal, R., Sastry, M., Dai, K., et al. (2011). Structure of HIV-1 gp120 V1/V2 domain with broadly neutralizing antibody PG9. Nature *480*, 336–343.

Otwinowski, Z., and Minor, W. (1997). Processing of X-ray diffraction data collected in oscillation mode. Methods Enzymol. *276*, 307–326.

Pauthner, M., Havenar-Daughton, C., Sok, D., Nkolola, J.P., Bastidas, R., Boopathy, A.V., Carnathan, D.G., Chandrashekar, A., Cirelli, K.M., Cottrell, C.A., et al. (2017). Elicitation of robust tier 2 neutralizing antibody responses in nonhuman primates by HIV envelope trimer immunization using optimized approaches. Immunity *46*, 1073–1088.e6.

Pettersen, E.F., Goddard, T.D., Huang, C.C., Couch, G.S., Greenblatt, D.M., Meng, E.C., and Ferrin, T.E. (2004). UCSF Chimera–a visualization system for exploratory research and analysis. J. Comput. Chem. *25*, 1605–1612.

Potter, C.S., Chu, H., Frey, B., Green, C., Kisseberth, N., Madden, T.J., Miller, K.L., Nahrstedt, K., Pulokas, J., Reilein, A., et al. (1999). Leginon: a system for fully automated acquisition of 1000 electron micrographs a day. Ultramicroscopy *77*, 153–161.

Pritchard, L.K., Harvey, D.J., Bonomelli, C., Crispin, M., and Doores, K.J. (2015). Cell- and protein-directed glycosylation of native cleaved HIV-1 envelope. J. Virol. *89*, 8932–8944.

Sanders, R.W., and Moore, J.P. (2017). Native-like Env trimers as a platform for HIV-1 vaccine design. Immunol. Rev. *275*, 161–182.

Sanders, R.W., Derking, R., Cupo, A., Julien, J.P., Yasmeen, A., de Val, N., Kim, H.J., Blattner, C., de la Peña, A.T., Korzun, J., et al. (2013). A next-generation cleaved, soluble HIV-1 Env trimer, BG505 SOSIP.664 gp140, expresses multiple epitopes for broadly neutralizing but not non-neutralizing antibodies. PLoS Pathog. *9*, e1003618.

Sanders, R.W., van Gils, M.J., Derking, R., Sok, D., Ketas, T.J., Burger, J.A., Ozorowski, G., Cupo, A., Simonich, C., Goo, L., et al. (2015). HIV-1 VACCINES. HIV-1 neutralizing antibodies induced by native-like envelope trimers. Science *349*, aac4223.

Scheres, S.H.W. (2015). Semi-automated selection of cryo-EM particles in RELION-1.3. J. Struct. Biol. *189*, 114–122.

Schrödinger, LLC (2015). The PyMOL molecular graphics system, version 1.8.

Sok, D., van Gils, M.J., Pauthner, M., Julien, J.-P., Saye-Francisco, K.L., Hsueh, J., Briney, B., Lee, J.H., Le, K.M., Lee, P.S., et al. (2014). Recombinant HIV envelope trimer selects for quaternary-dependent antibodies targeting the trimer apex. Proc. Natl. Acad. Sci. USA *111*, 17624–17629.

Stamatatos, L., Pancera, M., and McGuire, A.T. (2017). Germline-targeting immunogens. Immunol. Rev. *275*, 203–216.

Steichen, J.M., Kulp, D.W., Tokatlian, T., Escolano, A., Dosenovic, P., Stanfield, R.L., McCoy, L.E., Ozorowski, G., Hu, X., Kalyuzhniy, O., et al. (2016). HIV vaccine design to target germline precursors of glycan-dependent broadly neutralizing antibodies. Immunity *45*, 483–496.

Tabb, D.L., McDonald, W.H., and Yates, J.R., 3rd. (2002). DTASelect and Contrast: tools for assembling and comparing protein identifications from shotgun proteomics. J. Proteome Res. *1*, 21–26.

Voss, N.R., Yoshioka, C.K., Radermacher, M., Potter, C.S., and Carragher, B. (2009). DoG Picker and TiltPicker: software tools to facilitate particle selection in single particle electron microscopy. J. Struct. Biol. *166*, 205–213.

Walker, L.M., Phogat, S.K., Chan-Hui, P.-Y., Wagner, D., Phung, P., Goss, J.L., Wrin, T., Simek, M.D., Fling, S., Mitcham, J.L., et al.; Protocol G Principal

Investigators (2009). Broad and potent neutralizing antibodies from an African donor reveal a new HIV-1 vaccine target. Science *326*, 285–289.

Walker, L.M., Huber, M., Doores, K.J., Falkowska, E., Pejchal, R., Julien, J.-P., Wang, S.-K., Ramos, A., Chan-Hui, P.-Y., Moyle, M., et al.; Protocol G Principal Investigators (2011). Broad neutralization coverage of HIV by multiple highly potent antibodies. Nature *477*, 466–470.

Xu, T., Park, S.K., Venable, J.D., Wohlschlegel, J.A., Diedrich, J.K., Cociorva, D., Lu, B., Liao, L., Hewel, J., Han, X., et al. (2015). ProLuCID: An improved SEQUEST-like algorithm with enhanced sensitivity and specificity. J. Proteomics *129*, 16–24.

Zhang, K. (2016). Gctf: Real-time CTF determination and correction. J. Struct. Biol. *193*, 1–12.

Zheng, S.Q., Palovcak, E., Armache, J.-P., Verba, K.A., Cheng, Y., and Agard, D.A. (2017). MotionCor2: anisotropic correction of beam-induced motion for improved cryo-electron microscopy. Nat. Methods *14*, 331–332.

Cell Reports, Volume *23*

Supplemental Information

Co-evolution of HIV Envelope and Apex-Targeting Neutralizing Antibody Lineage Provides Benchmarks for Vaccine Design

Kimmo Rantalainen, Zachary T. Berndsen, Sasha Murrell, Liwei Cao, Oluwarotimi Omorodion, Jonathan L. Torres, Mengyu Wu, Jeffrey Umotoy, Jeffrey Copps, Pascal Poignard, Elise Landais, James C. Paulson, Ian A. Wilson, and Andrew B. Ward

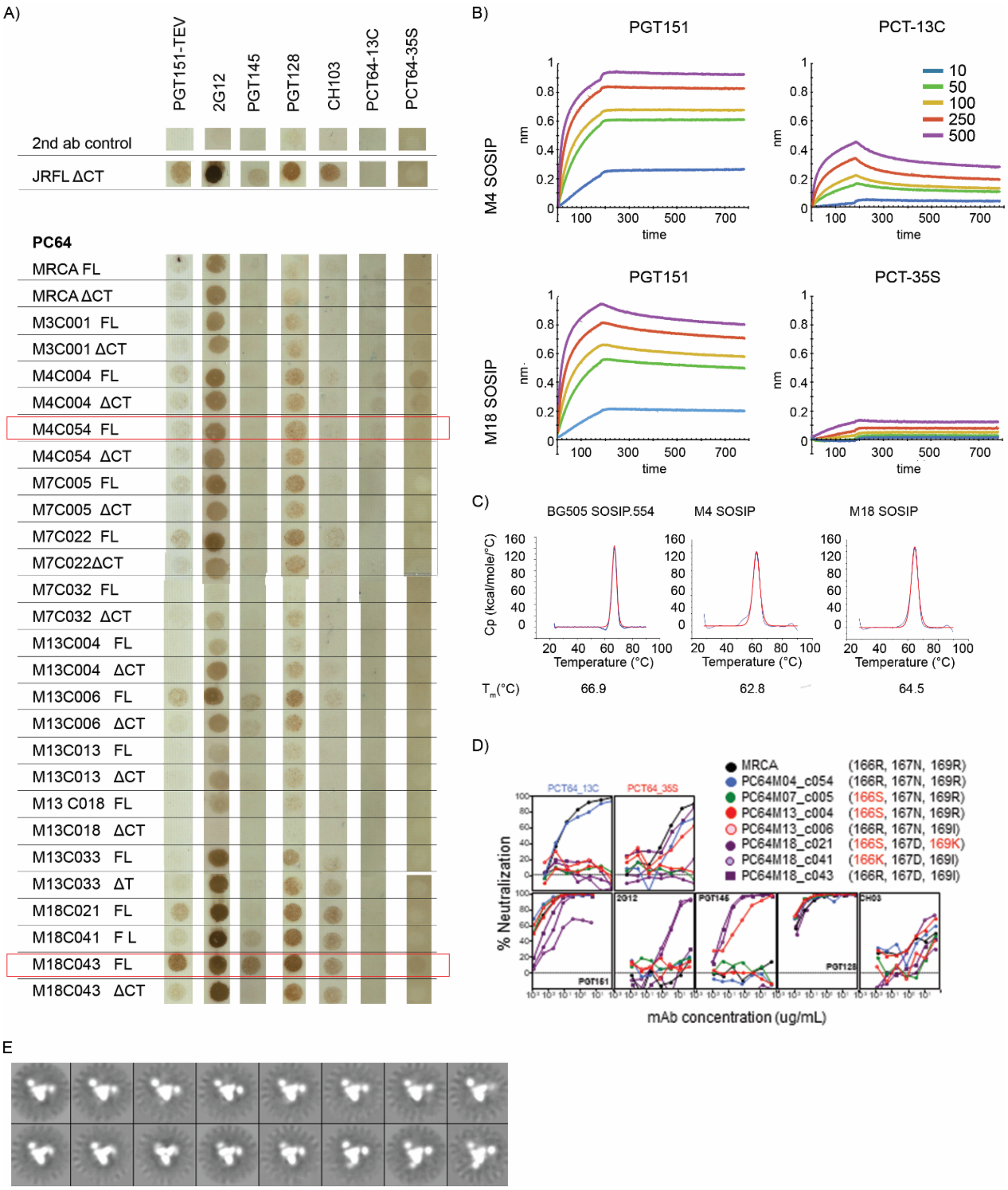

Supplemental Figure 1. **Screening metrics for representative Early and Late timepoint Env and autologous antibodies. Related to Figure 1.** (A) C-terminally truncated (ΔCT) and full-length (FL) Env constructs expression was measured by relative antibody staining in a dotblot assay. Conformationally sensitive control antibodies PGT151 and PGT145 were used to measure expression levels of well-formed trimers. Highest expressing clone PC64M18C043 (Late Env) was selected for high resolution cryo-EM studies and earlier time point clone PC64M4C054 as the representative early Env time point clone. (B) Octet analysis of Early and Late SOSIP binding to PGT151 and autologous Early and Late antibodies. Inset shows the concentration of antibody in nM. (C) Melting point of BG505 SOSIP and PC64 Envs used in this study in a DSC assay. (D) Immunogenic profiling of the selected clones against heterologous antibodies. (E) Negative stain 2D classes of unliganded Late SOSIP.

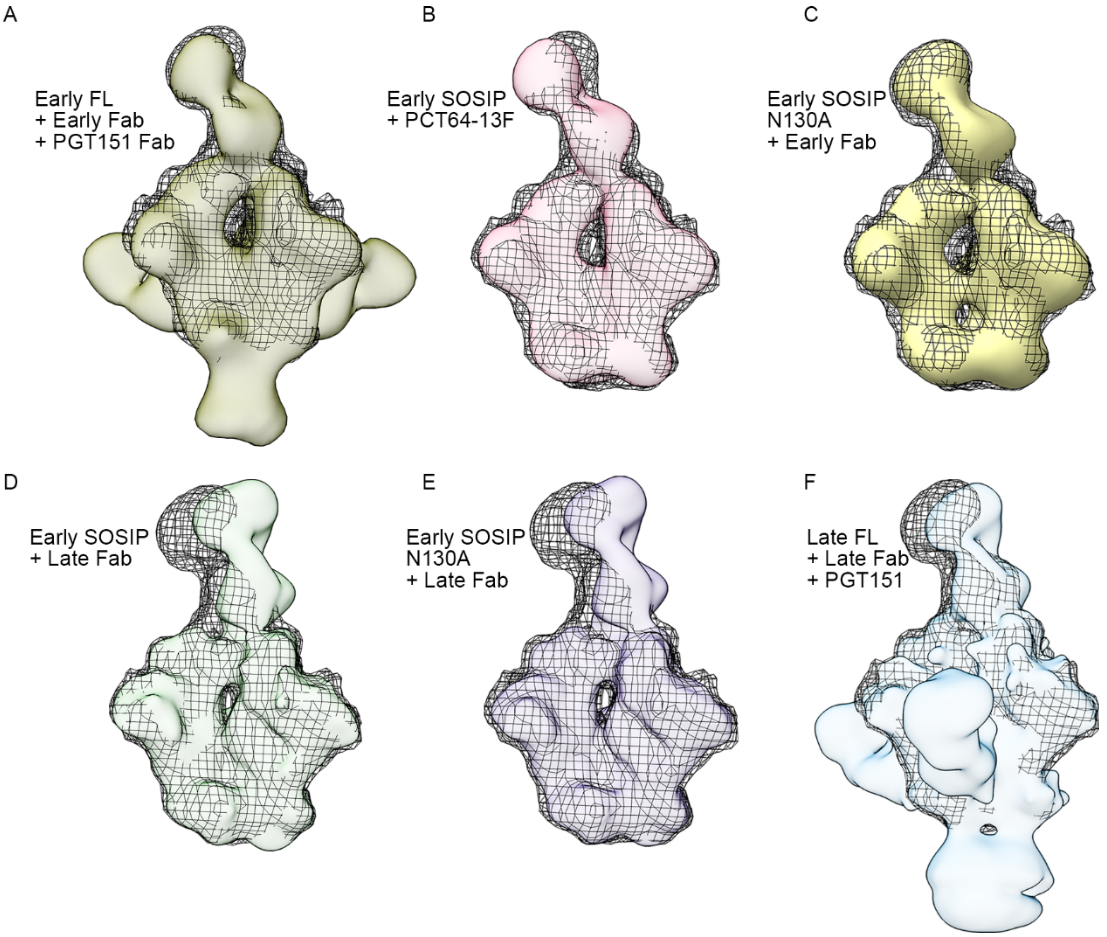

Supplemental Figure 2. Antibody binding angle comparisons. Related to Figures 1 and 2. Autologous complexes are shown in different colors. In each case the Early SOSIP – Early Fab complex (grey mesh) is overlaid as a reference. The binding angle was approx. 20° steeper in all complexes with the Late antibody. A, B and C were reconstructed from negative stain EM data. For comparison, D, E and F were gaussian filtered from higher resolution cryo-EM maps to ~20Å to match the resolution of the negative stain reconstructions.

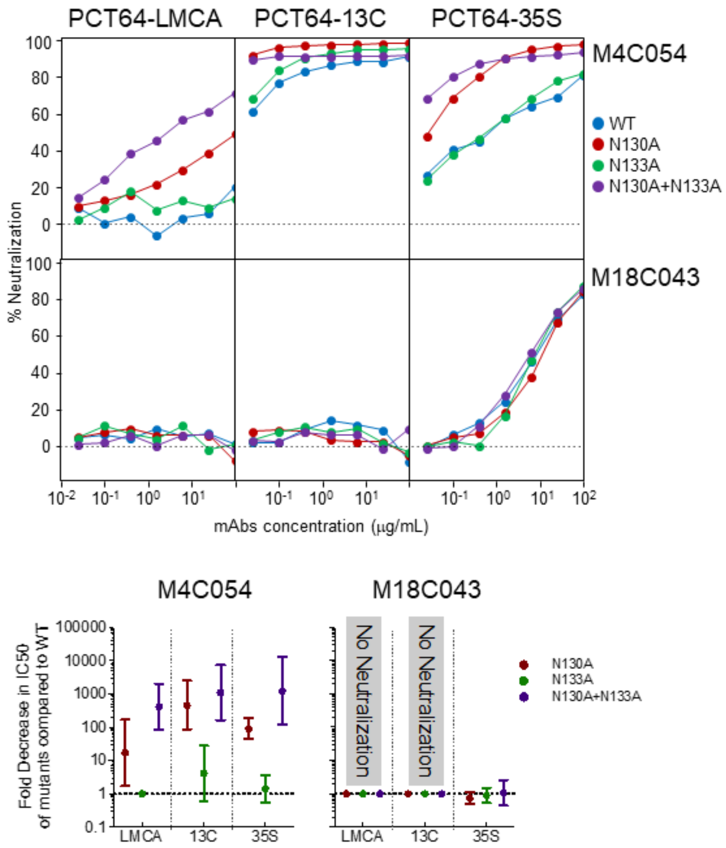

Supplemental Figure 3. Effect of N130- and N133 glycan removal on PC64 early and late autologous neutralization. **Related to Figures 3 and 4.** (A) Autologous neutralization of the WT and mutants Early Env (top) and Late Env (bottom) pseudotyped PC64 viruses by titrated amounts of the indicated PCT64 mAbs, averaged across 8 independent experiments. (B) Geometric mean with geometric SD (N=8) of fold decrease in neutralization IC50 (Ab concentration in µg/mL that represents 50% loss of infectivity) of mutants compared to the corresponding WT virus.

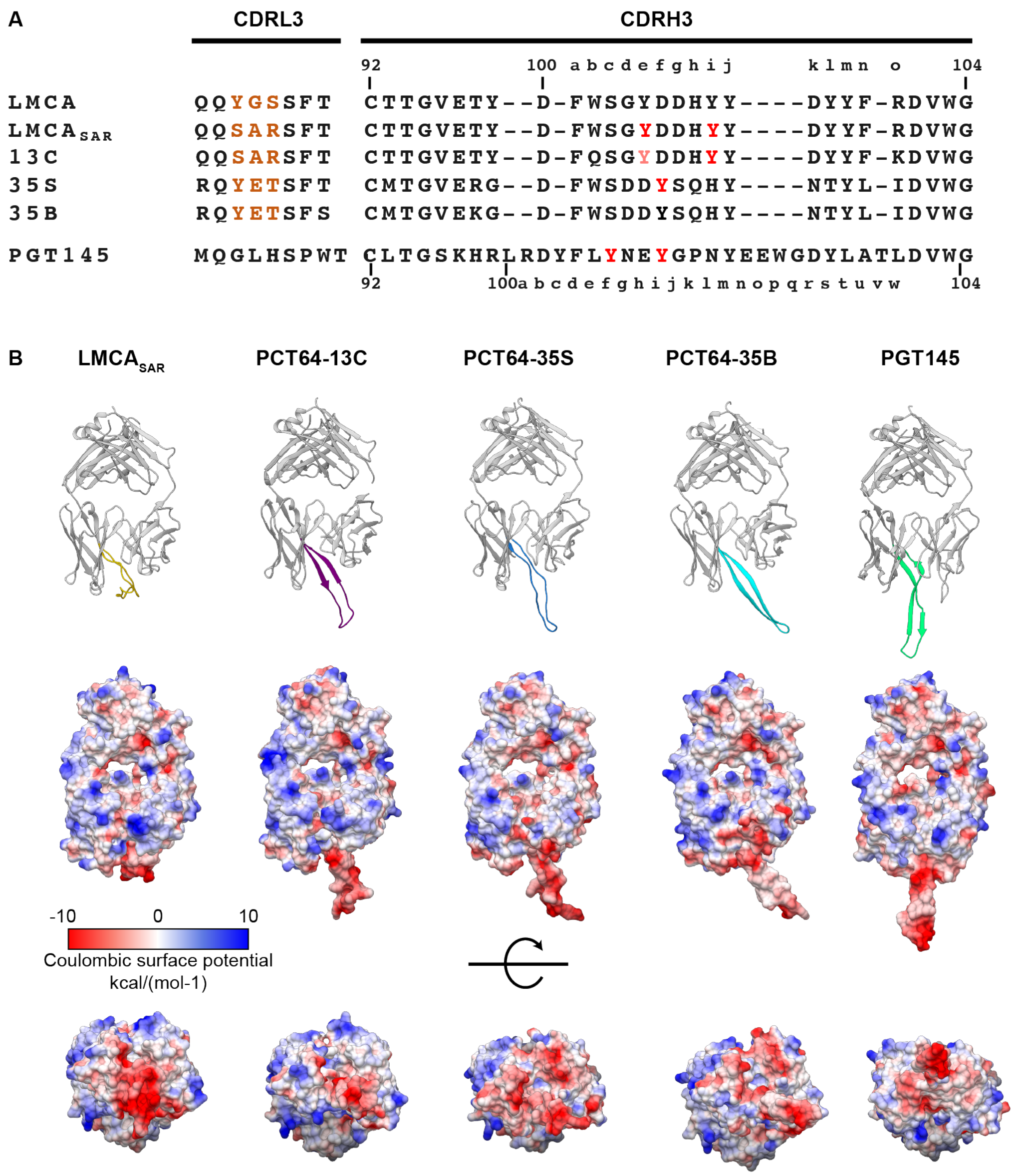

Supplemental Figure 4. Structure and CDR3 sequences of PCT64 and PGT145 antibodies. Related to Figure 1. (A) Alignment of Fab CDRL3 and CDRH3 adapted from T-Coffee (Kabat numbering indicated for CDRH3). Confirmed sulfated tyrosines indicated by red font. (B) Cartoon and Electrostatic surface potential representation of: LMCA$_{SAR}$, PCT64-13C, PCT64-35S, PCT64-35B (5FEH), PGT145 (3U1S).

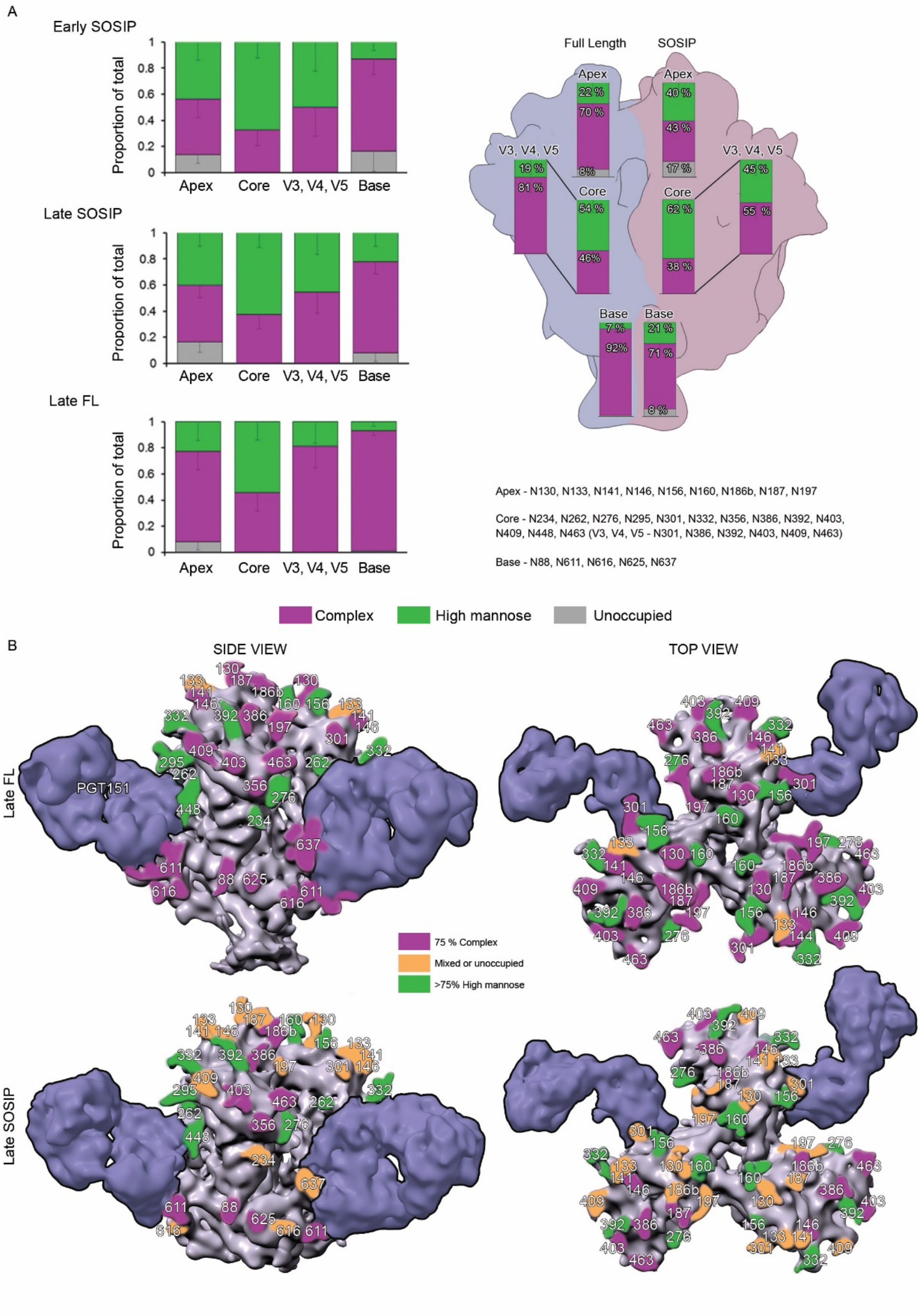

Supplemental Figure 5. Grouping of glycan processing based on their location on Env in FL versus SOSIP. Related to Figure 6. (A) Glycans were grouped into three regions (apex, core, base or V3/V4/V5) based on their location in the trimer as indicated. Proportion of high mannose (green), complex type (purple) or unoccupied (grey) glycosites. (B) Detailed mapping of the glycosylation differences on the surface of corresponding cryo-EM density maps with two PGT151 Fabs bound per trimer.

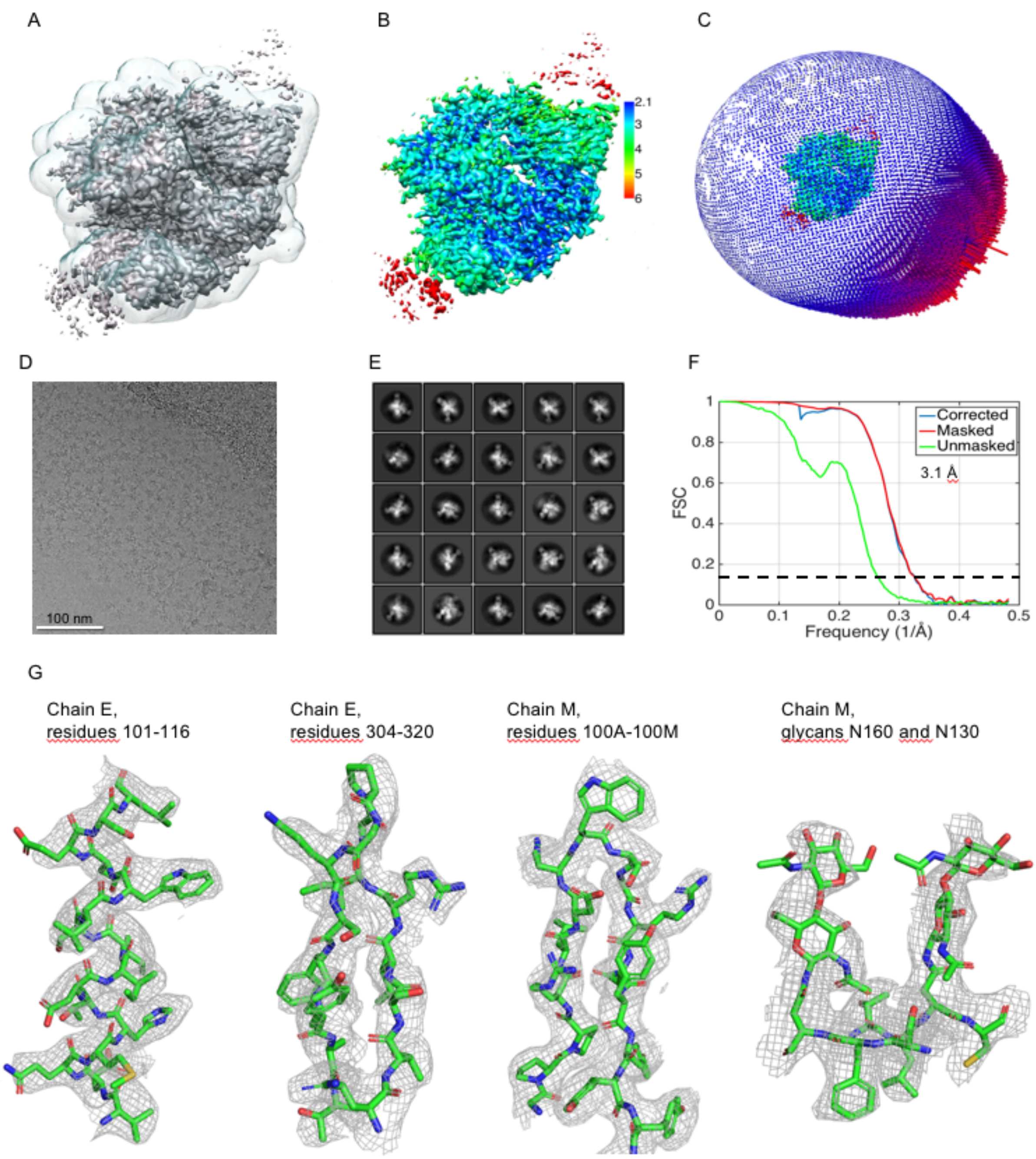

Supplemental Figure 6. Supporting data for Cryo-EM maps. Related to supplemental experimental procedures: EM data processing. (A) Late FL EM density map (grey) with soft binary mask (transparent blue) used during refinement, classification, and sharpening. (B) Local resolution map (ResMap). (C) Anglular distribution 3D histogram. (D) Representative image of aligned raw micrographs. (E) 2D Classes. (F) Fourier-Shell-Correlation (FSC) plot with dashed lined indicating FSC=0.143. (E) Representative density snap shots of the Late FL map.

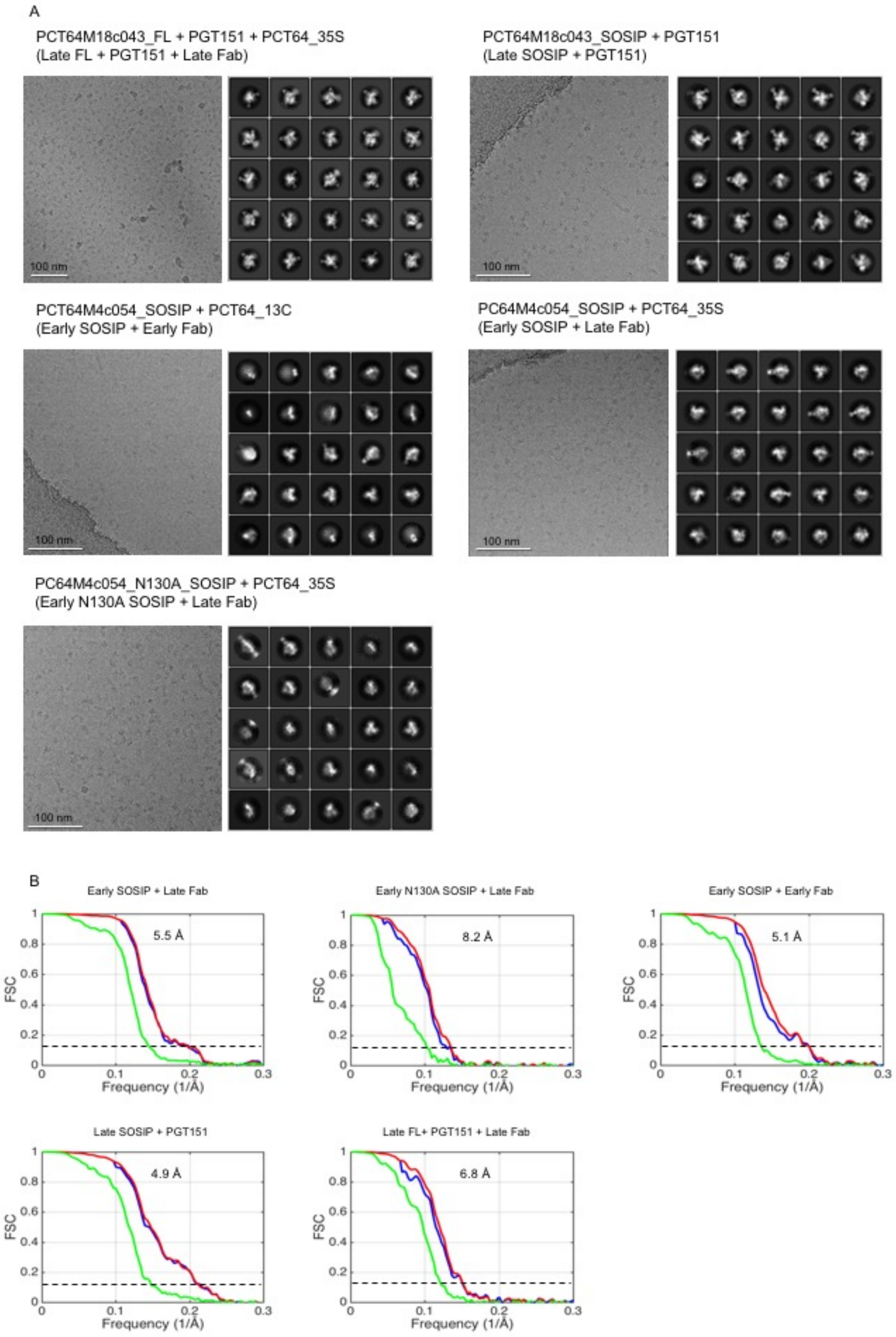

Supplemental Figure 7. Supporting data for Cryo-EM maps. Related to supplemental experimental procedures: EM data processing. (A) Representative images of aligned raw micrographs and 2D classes. (B) FSC plots for 5 cryo-EM maps.

SUPPLEMENTAL FIGURES AND TABLES

Supplemental Table 1. Cryo-EM samples and negative stain, imaging conditions and processing statistics. Related to Figure 1.

**Env**	PC64M18C043 FL (Late FL)	PC64M18C043 FL (Late FL)	PC64M18C043 SOSIP (Late SOSIP)	PC64M4C054 SOSIP (Early SOSIP)	PC64M4C054 SOSIP (Early SOSIP)	PC64M4C054 N130A SOSIP (Early N130A SOSIP)	PC64M4C054 (Early FL)	PC64M4C054 N130A SOSIP (Early N130A SOSIP)	PC64M4C054 (Early SOSIP)
**Ligands**	+ PGT151 Fab	+ PGT151 Fab<br>+ PCT64-35S (Late Fab)	+ PGT151 Fab	+ PCT-13C Fab (Early Fab)	+PCT-35S Fab (Late Fab)	+PCT-35S Fab (Late Fab)	+ PGT151 Fab + PCT13C Fab (Early Fab)	+PCT13C Fab(Early Fab)	+PCT13F Fab
**Sample type**	Cryo-EM	Cryo-EM Cont. Carbon	Cryo-EM	Cryo-EM	Cryo-EM	Cryo-EM	Negative Stain EM	Negative Stain EM	Negative Stain EM
**Data collection**									
Microscope	FEI Titan Krios	FEI Talos Arctica	FEI Titan Krios	FEI Titan Krios	FEI Titan Krios	FEI Titan Krios	FEI Tecnai Spirit	FEI Tecnai Spirit	FEI Tecnai Spirit
Voltage (kV)	300	200	300	300	300	300	120	120	120
Detector	Gatan K2 Summit	Gatan K2 Summit	Gatan K2 Summit	Gatan K2 Summit	Gatan K2 Summit	Gatan K2 Summit	TemCam F416	TemCam F416	TemCam F416
Recording mode	counting	counting	counting	counting	counting	counting	linear	linear	linear
Magnification (incl. post-magnification)	48,534	34,247	48,534	48,534	48,534	48,534	74,146	74,146	74,146
Movie micrograph pixel size (Å)	1.03	1.46	1.03	1.03	1.03	1.03	2.05	2.05	2.05
Dose rate (e^-/[(camera pixel)*s])	10	10	10	10	7.2	8	420	323	323

Number of frames per movie micrograph	40	32	43	43	38	33	NA	NA	NA
Frame exposure time (ms)	200	250	200	200	250	250	250	325	325
Movie micrograph exposure time (s)	8	8	8.6	8.6	9.5	8.25	NA	NA	NA
Total dose ($e^-/Å^2$)	75.4	37.5	81.1	81.1	64.5	62.2	25	25	25
Defocus range (µm)	-0.5-3.5um	-0.5-4um	-0.2-0.5um	-0.5-3.5um	-0.5-4um	-0.5-4um	-0.85-2.1um	-0.9-2.7um	-1.0-2.7um
**EM data processing**									
Number of movie micrographs	4039	980	1555	2006	2007	867	776	222	370
Number of molecular projection images in map	23,6179	60,093	34,864	29,672	45,661	25,133	2311	2395	8633
Symmetry	C1	C1	C1	C1	C1	C1	C1	C1	C1
Map resolution (FSC 0.143 (CRYO) or 0.5 (NS); Å)	3.1	6.8	4.9	5.1	5.5	8.2	30	30	30
Map sharpening B-factor ($Å^2$)	-70	-427	-186	-250	-235	-568	NA	NA	NA
**EMD accession code**	EMD-7858	EMD-7859	EMD-7860	EMD-7863	EMD-7865	EMD-7866	EMD-7861	EMD-7864	EMD-7862

Supplemental Table 2. Data collection parameters and refinement statistics for the Fab crystal structures and for the PC64M18C043 FL cryo-EM structure. Related to Figure 1.

**Name**	PCT64-LMCA ($CDRL3_{SAR}$)	PCT64-13C (Early Fab)	PCT64-35S (Late Fab)	PC64M18C043 FL (Late FL) +PGT151 Fab
**Data Collection**	SSRL 12-2	APS 23-ID-D	APS 23-ID-D	Titan Krios
Wavelength, Å	0.9795	1.0332	1.0332	n/a
Space group	$P2_12_12_1$	$P2_1$	$P2_12_12_1$	n/a
Unit cell a, b, c (Å)	84.20 87.16 142.57	43.09 68.85 78.50	96.78 98.13 100.56	n/a
α, β, γ (°)	90 90 90	90 94.27 90	90 90 90	n/a
Fabs per ASU	2	1	2	n/a
Resolution (Å)*	50.0 – 2.70 (2.75 – 2.70)	50.0 – 1.64 (1.68 -1.64)	50.0 – 2.43 (2.47– 2.43)	3.1
Completeness*	93.4 (68.7)	99.4 (89.3)	99.7 (95.2)	n/a
Redundancy*	5.9 (3.8)	5.9 (3.7)	11.4 (4.7)	n/a
No. total reflections	162,629	326,819	418,936	n/a
No. unique reflections	27,556 (998)	55,491 (2478)	36,695 (1716)	n/a
I/σ*	12.4 (2.3)	19.6 (2.8)	18.3 (12.3)	n/a
R_{sym}*	0.12 (0.47)	0.10 (0.49)	0.15 (0.62)	n/a
R_{pim}*	0.05 (0.25)	0.04 (0.24)	0.04 (0.26)	n/a
$CC_{1/2}$*	0.98 (0.91)	0.98 (0.82)	0.95 (0.75)	n/a
**Refinement statistics**				
Resolution (Å)	43.6 – 2.70	43.0 - 1.64	49.1 - 2.43	3.1
No. reflections total/R_{free}	27,451/1,340	52,835/2634	34,715/1,872	n/a
R_{cryst}‡/R_{free}§	24.2/26.8	16.9/19.0	20.0/24.5	n/a
RMSD bond length (Å)	0.002	0.006	0.006	0.008
RMSD bond angles (°)	0.49	0.89	0.87	1.282
Protein atoms/solvent atoms	6769/42	3578/515	6777/180	19390/ n/a
Average B-value ($Å^2$) overall/protein/solvent	75/75/51	25/24/33	40/40/36	n/a
Fab 1 B-value ($Å^2$)	62	24	35	n/a
Fab 2 B-value ($Å^2$)	88	n/a	44	n/a
Wilson B-value ($Å^2$)	53	19	35	n/a
Ramachandran Favored%	97.0	98.4	98.1	91.56
Ramachandran Outliers%	0.0	0.0	0.0	0.09
MolProbity score	1.42	0.98	1.13	1.79
ClashScore	4.79	2.12	3.15	5.41
EMringer score	n/a	n/a	n/a	3.43
PDB ID	6CA9	6CA7	6CA6	6DCQ

* Values in parentheses are for highest-resolution shell.

SUPPLEMENTAL VIDEO LEGENDS

Supplemental Video 1. Related to Figure 1. The effect of PGT151 Fab on quaternary structure of Late SOSIP versus Late FL. Both Cryo-EM models were aligned to high resolution reconstruction of the Late ectodomain. At ~6Å resolution a slight change in the orientation of PGT151 Fab was observed.

Supplemental Video 2. Related to Figure 2. Rigidification of CDRH3 and maturation of the antibody approach angle. Rigidification of CDRH3 is shown by morphing the morph between Early SOSIP complexed with either Early or Late Fab. The corresponding crystal structure with the CDRH3 loop fitted in the cryo-EM reconstruction is simultaneously morphed showing the deeper reach of the later antibody. Concomitantly with this, the angle matures to ~20° steeper.

Supplemental Video 3 Related to Figure 3. Apex glycans in Late FL participating in binding of the Late Fab.

Supplemental Experimental Procedures

Expression and folding screening of full length and ΔCT clones

A dotblot assay was developed for screening the expression of full-length and C-terminally truncated clones. A panel of 28 full-length and C- terminal deletion constructs from donor 64 were screened using bnAbs targeting different regions of Env as probes (Figure S1). 750ng of Env DNA and 187.5ng of Furin DNA was mixed with 2.8µg of PEI MAX in 150µl of OPTI-MEM medium in 24 well plates. Plates were sealed with gas-permeable plate seals, vortexed and incubated for 20 min at room temperature prior to addition of 3ml of 293F suspension cells at 1.3 – 1.5 million cells/ml. Cell density at the point of transfection, expression time and incubation conditions were optimized for 24-well plate format and tested for scalability with 25mL and 1L formats. Transfected cells were grown in +37°C, 80% humidity and 8% CO^2 for three days. Plate shaker was set to 500 rpm for 24 well format and to 130 rpm for 25mL and 1L formats. Cell density at the point of harvest (3 days post transfection) was between 4.5 and 6.5 million cells/ml and cell viability >80% in both 24-well, and up-scaled expressions.

Cells were harvested in 24-well plates for 15 min at 1 000 rcf, washed once with 1 ml cold PBS and either resuspended to cold PBS or lysed with TBS containing 0.5% triton X-100 (TBS-T). Suspensions were transferred to 96-well plates to allow application on PVDF membranes with 12-channel pipette. PVDF membranes were pre-wetted with 100% methanol for 20 sec, rinsed briefly with water, and soaked in TBST. Membranes were placed on stack of dry Whatman no. 3 paper followed by a filter paper pre-soaked with TBST to make sure that no air was trapped between the membrane and filter paper. Samples were applied using multichannel pipette with three to five µL of suspension per dot, followed by 20-minute incubation. Membranes were then blocked with PBS containing 5% (w/v) milk powder for 30 minutes in room temperature. Primary antibody was added at 1:1000 – 1:20 000 dilution, depending on the primary antibody, and incubated for 1h in room temperature. Membranes were then washed 3 times 5 minutes with PBS + 0.1% tween-20 (PBST) before the addition of secondary antibody (anti-human IgG, HRP conjugated) and incubated 1h at room temperature, followed by washing 3 times 5 minutes with PBST. Detection was done using colorimetric reaction (CN/DAB Substrate Kit, Thermo Scientific #34000).

Differential scanning calorimetry (DSC)

The thermal stability of the Early and Late SOSIP constructs was measured using a MicroCal VP-capillary DSC calorimeter (Malvern). SOSIP samples were buffer exchanged into phosphate-buffered saline (PBS, pH 7.4) and adjusted to a final concentration of 0.3 mg/mL. Thermal denaturation of each SOSIP sample was probed at a scan rate of 90°C/hr. Normalization and baseline subtraction were performed prior to data analysis using the MicroCal Automatic Analysis program. A final curve was obtained by fitting the data using a non-two-state model.

Biolayer interferometry (BLI)

Biolayer interferometry was used to assess kinetics of Early and Late SOSIP binding to PGT151 antibody and to autologous Early and Late antibodies. Binding measurements were performed using an Octet RED96 instrument (fortéBio, Pall Life Sciences). Assays were conducted at 25°C or 37°C in solid black 96-well plates (Geiger Bio-One) with constant agitation at 1,000 rpm. Antibody (25 μg/mL) in 1X Kinetics Buffer (PBS pH 7.4, 0.01% [w/v] BSA, 0.002% [v/v] Tween 20) was loaded onto anti-human Fc Capture Biosensors (AHC) to threshold binding (approx. 2 nm), followed by a 60 second baseline acquisition in 1X Kinetics Buffer. A concentration gradient of testing antigen (500 nM, 250 nM, 100 nM, 50 nM, 10 nM) was applied for 180 seconds association, followed by measurement of dissociation in 1X Kinetics Buffer for 600 seconds. Octet data were processed using the fortéBio data acquisition program (v.8.1). Experimental data were fitted for PGT151 and Early antibodies using a global fit 1:1 model to determine K_D values. Baseline drift correction was performed by subtraction of the measurement for a sensor loaded with antibody dipped into 1X Kinetics buffer without antigen.

Neutralization assay

Human embryonic kidney (HEK)-derived 293T and HeLa-derived TZM-bl cells were maintained in complete Dulbecco's Modified Eagle Medium containing high-glucose Dulbecco's Modified Eagle Medium (DMEM, Thermo Fisher), 1X Penicillin-Streptomycin (Thermo Fisher) and 10% fetal bovine serum (Gemini Bio Products) at 37°C / 5% CO2. Monoclonal antibody neutralizing activity was assessed using single round of replication in TZM-bl target cells in the absence of DEAE-dextran, as described previously (Landais et al., PLoS Pathog. 2016). Briefly, WT and mutant pseudoviruses were produced by co-transfection of HEK 293-T with an Env-expressing plasmid and an Env-deficient genomic backbone plasmid (pSG3ΔEnv). Env mutagenesis was performed using the Quikchange site-directed mutagenesis kit (Agilent Technologies).

EM data processing

All direct detector movies were aligned and dose-weighted using MotionCor2 (Zheng et al., 2017) (Figure S6, S7). Contrast transfer function (CTF) parameters were estimated from non-dose weighted micrographs using GCTF (Zhang, 2016). Micrographs with estimated resolutions or CTF fits below a certain threshold were removed from the data set. Projection images of Env were selected from each micrograph automatically with either Relion template picking using either templates from previous data sets or a Gaussian blob, or using DoGPicker (Voss et al., 2009). If the micrographs were collected around the edges of carbon holes, EMHP was used to mask out carbon surfaces and filter particle picks (Berndsen et al., 2017). All subsequent downstream single-particle operations were performed with gpu-accelerated Relion/2.0 (Kimanius et al., 2016). As a first step, 4x binned particles were extracted from dose-weighted micrographs and one to three rounds of reference-free 2D classification were performed followed by subset selection and particle re-centering to select for high-resolution classes and remove false positives. These steps

generally remove 10-20% of initial particle picks. Once the data set was deemed sufficiently clean, unbinned particles were extracted and sent through one initial round of unmasked 3D auto-refinement against a reference cryo-EM map of an unliganded HIV-1 Env. Refined Euler angles were used as a starting point for one or more rounds of 3D classification with limited angular sampling. The number of classes in each round of 3D classification varied substantially depending on the specifics of each data set. Homogeneous subsets of 3D classes were selected and particles were re-centered and re-extracted for a final time. At this point, 3D binary masks were generated from heavily lowpass filtered 3D classes and used for any additional rounds of classification and refinement. 3D auto refinement was performed on the homogeneous subsets of particles selected during 3D classification while applying a binary mask. After 3D auto-refinement, each map was sharpened with the mask used in refinement, commonly applying a B-factor generated automatically in Relion. The 3.1 Å Late FL + PGT151 map was sharpened with an ad hoc B-factor of -70 after auto B-factors were determined to be too low by visual inspection. All B-factors used for sharpening are presented in table S1 and all binary masks are deposited to the EMDB, along with unsharpened half maps and final sharped maps. Additional rounds of 3D classification with restricted sampling were performed if the data set was large or heterogeneous, often using high values of the *tau_fudge* (10-20) parameter in Relion, with higher values used for higher resolution data sets. When resolution gains were no longer achieved through classification or classes were deemed to be homogeneous, a final round of 3D auto refinement followed by sharpening was performed. For the 3.1 Å reconstruction of Late FL, an additional step of per-particle CTF estimation was performed with GCTF. CTF parameters were estimated for each particle and used in subsequent rounds of refinement and classification resulting in a 0.3 Å higher resolution reconstruction than was achieved when using CTF parameters estimated from whole micrographs (Figure S6).

Supplemental video editing

Assembly of videos was started by aligning Cryo-EM reconstructions to high resolution reconstruction of Late FL ectodomain using UCSF Chimera. CDRH3 loops of Early and Late Fabs were adjusted according to their corresponding complexed densities using real space refine function in COOT prior to fitting in Cryo-EM maps using Fit In Map function in UCSF Chimera. Morphs between reconstructions in chimera recorded as separate clips in UCSF chimera and assembled in Adobe premiere Pro (Adobe).

Supplemental references

Zheng, S.Q., Palovcak, E., Armache, J.-P., Verba, K.A., Cheng, Y., and Agard, D.A. (2017). MotionCor2: anisotropic correction of beam-induced motion for improved cryo-electron microscopy. Nat. Methods *14*, 331–332.

Zhang, K. (2016). Gctf: Real-time CTF determination and correction. J Struct Biol *193*, 1–12.

Voss, N.R., Yoshioka, C.K., Radermacher, M., Potter, C.S., and Carragher, B. (2009). DoG Picker and TiltPicker: software tools to facilitate particle selection in single particle electron microscopy. J Struct Biol *166*, 205–213.

Berndsen, Z., Bowman, C., Jang, H., and Ward, A.B. (2017). EMHP: An accurate automated hole masking algorithm for single-particle cryo-EM image processing. Bioinformatics.

Kimanius, D., Forsberg, B.O., Scheres, S.H., and Lindahl, E. (2016). Accelerated cryo-EM structure determination with parallelisation using GPUs in RELION-2. Elife *5*, 19.
